# Ectomycorrhizal ecology is imprinted in the genome of the dominant symbiotic fungus *Cenococcum geophilum*

**DOI:** 10.1038/ncomms12662

**Published:** 2016-09-07

**Authors:** Martina Peter, Annegret Kohler, Robin A. Ohm, Alan Kuo, Jennifer Krützmann, Emmanuelle Morin, Matthias Arend, Kerrie W. Barry, Manfred Binder, Cindy Choi, Alicia Clum, Alex Copeland, Nadine Grisel, Sajeet Haridas, Tabea Kipfer, Kurt LaButti, Erika Lindquist, Anna Lipzen, Renaud Maire, Barbara Meier, Sirma Mihaltcheva, Virginie Molinier, Claude Murat, Stefanie Pöggeler, C. Alisha Quandt, Christoph Sperisen, Andrew Tritt, Emilie Tisserant, Pedro W. Crous, Bernard Henrissat, Uwe Nehls, Simon Egli, Joseph W. Spatafora, Igor V. Grigoriev, Francis M. Martin

**Affiliations:** 1 Swiss Federal Research Institute WSL, Forest Dynamics, Zuercherstrasse 111, 8903 Birmensdorf, Switzerland; 2 INRA, UMR INRA-Université de Lorraine ‘Interactions Arbres/Microorganismes', Laboratoire d'Excellence ARBRE, INRA-Nancy, 54280 Champenoux, France; 3 US Department of Energy Joint Genome Institute (JGI), Walnut Creek, California 94598, USA; 4 Microbiology, Department of Biology, Utrecht University, 3508 TB Utrecht, The Netherlands; 5 University of Bremen, Botany, Leobenerstr. 2, 28359 Bremen, Germany; 6 CBS-KNAW Fungal Biodiversity Centre, Uppsalalaan 8, 3584 CT Utrecht, The Netherlands; 7 Institute of Microbiology and Genetics, Department of Genetics of Eukaryotic Microorganisms, Georg-August-University Göttingen, 37077 Göttingen, Germany; 8 Göttingen Center for Molecular Biosciences (GZMB), Georg-August-University Göttingen, 37077 Göttingen, Germany; 9 Department of Ecology and Evolutionary Biology, University of Michigan, Ann Arbor, Michigan 48109 USA; 10 Centre National de la Recherche Scientifique, UMR 7257, F-13288 Marseille, France; 11 Architecture et Fonction des Macromolécules Biologiques, Aix-Marseille University, F-13288 Marseille, France; 12 INRA, USC 1408 AFMB, F-13288 Marseille, France; 13 Department of Biological Sciences, King Abdulaziz University, Jeddah 21589, Saudi Arabia; 14 Department of Botany and Plant Pathology, Oregon State University, Corvallis, Oregon 97331 USA

## Abstract

The most frequently encountered symbiont on tree roots is the ascomycete *Cenococcum geophilum,* the only mycorrhizal species within the largest fungal class Dothideomycetes, a class known for devastating plant pathogens. Here we show that the symbiotic genomic idiosyncrasies of ectomycorrhizal basidiomycetes are also present in *C. geophilum* with symbiosis-induced, taxon-specific genes of unknown function and reduced numbers of plant cell wall-degrading enzymes. *C. geophilum* still holds a significant set of genes in categories known to be involved in pathogenesis and shows an increased genome size due to transposable elements proliferation. Transcript profiling revealed a striking upregulation of membrane transporters, including aquaporin water channels and sugar transporters, and mycorrhiza-induced small secreted proteins (MiSSPs) in ectomycorrhiza compared with free-living mycelium. The frequency with which this symbiont is found on tree roots and its possible role in water and nutrient transport in symbiosis calls for further studies on mechanisms of host and environmental adaptation.

## Abstract (web summary)

The ascomycete *Cenococcum geophilum* is a beneficial mycorrhizal symbiont found frequently on tree roots. Here the authors use comparative genomics and transcriptomics to define genomic signatures that differentiate the beneficial *C. geophilum* from its saprotrophic and pathogenic relatives.

## Graphical abstract

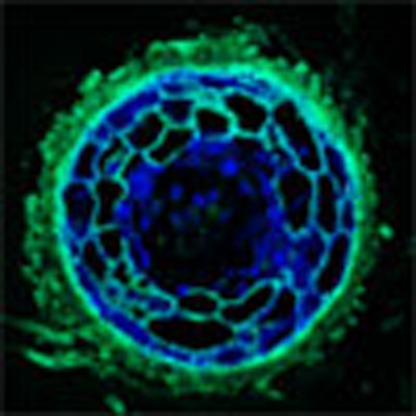

The ‘symbiosis molecular toolbox' of a dozen of ectomycorrhizal (ECM) basidiomycetes has recently been characterized by large-scale comparative genomics^1^. These analyses have shown that through contraction and loss of major gene families such as plant cell wall-degrading enzymes (PCWDE), secreted invertases, and toxin synthesis genes, ECM symbionts have become highly reliant upon the availability of a continuous flux of photoassimilates from their host plant, while preserving plant cell integrity by avoiding the release of PCWDE and toxins^1^,^2^. Transcript profiling of several mycorrhizal interactions revealed that a significant proportion of the symbiosis-upregulated genes are taxon-specific, restricted to a single mycorrhizal species^1^. Within these taxon-specific orphan genes, symbiotic effectors such as mycorrhiza-induced, small secreted protein (MiSSP7), suppress root defense responses and alter root metabolism to accommodate the intruding hyphae and feed them with nutrients as observed for plant pathogens^3^. In contrast to basidiomycetes, only two genomes of ascomycetous ECM fungi, the Périgord black truffle (*Tuber melanosporum*) and the Hart's truffle (*Elaphomyces granulatus*) have been published so far^2^,^4^. The ascomycete *Cenococcum geophilum* Fr. is the most common and globally abundant ECM fungus and often dominates the root systems of trees in the arctic, temperate and subtropical zones and particularly in extreme environments^5^,^6^. It forms a characteristic melanized mantle around root tips with darkly pigmented, stringy hyphae emanating in the surrounding soil (Fig. 1b). The ECM tips are highly resistant to desiccation^7^ and are strikingly abundant during soil drought conditions when other ECM species decline, suggesting an important role in drought resistance and resilience of host trees^5^,^8^. Although being ubiquitous, the biology of *C. geophilum* is poorly understood. It forms sclerotia as resistant propagules, but no spore-producing structures are known. Studies of fine-scale genetic diversity in *C. geophilum* populations revealed a high level of sequence polymorphism consistent with the occurrence of recombination mechanisms, suggesting that the fungus is reproducing sexually in nature^9^,^10^,^11^.

The ECM association has arisen repeatedly from ancestral saprotrophs in most clades during fungal evolution leading to a surprisingly similar symbiotic organ and mutualistic relationship with host plants^1^. *C. geophilum* (Mytilinidiales, Gloniaceae) is the only ECM symbiont that evolved within the clade of Dothideomycetes^12^, the largest class of Ascomycota in which sequenced pathogens and saprotrophs show a large complement of genes coding for secondary metabolism (SM) and plant-cell-wall degrading enzymes (PCWDE)^13^. Therefore possibly more of these genes are present in *C. geophilum* than in other ECM fungi, which might have consequences on the fungal/host interaction.

Here we aimed to decipher which features of the mycorrhizal lifestyle and function are imprinted in the genome and symbiotic transcriptome of *C. geophilum* and to compare them with those of the ECM basidiomycetes. We sequenced the genomes of *C. geophilum* and two closely related saprotrophic Dothideomycetes in the Mytilinidiales, *Glonium stellatum* and *Lepidopterella palustris,* and compared them with other fungi having saprotrophic, pathogenic or mycorrhizal lifestyles. As most ECM fungi, *C. geophilum* has a large genome of 178 megabases due to the proliferation of transposable elements. This genome encodes a lower set of genes coding for PCWDEs compared with saprotrophic and pathogenic Dothideomycetes, although its arsenal of PCWDEs and toxin synthesis genes is larger than those of ECM basidiomycetes. Transcript profiling of ECM root tips indicate a striking upregulation of water channels, sugar transporters and MiSSPs. These findings call for further studies on the mechanisms of host and environmental adaptation during symbiosis.

## Results

### Genome assemblies and phylogenomic placement

**Figure 1:**
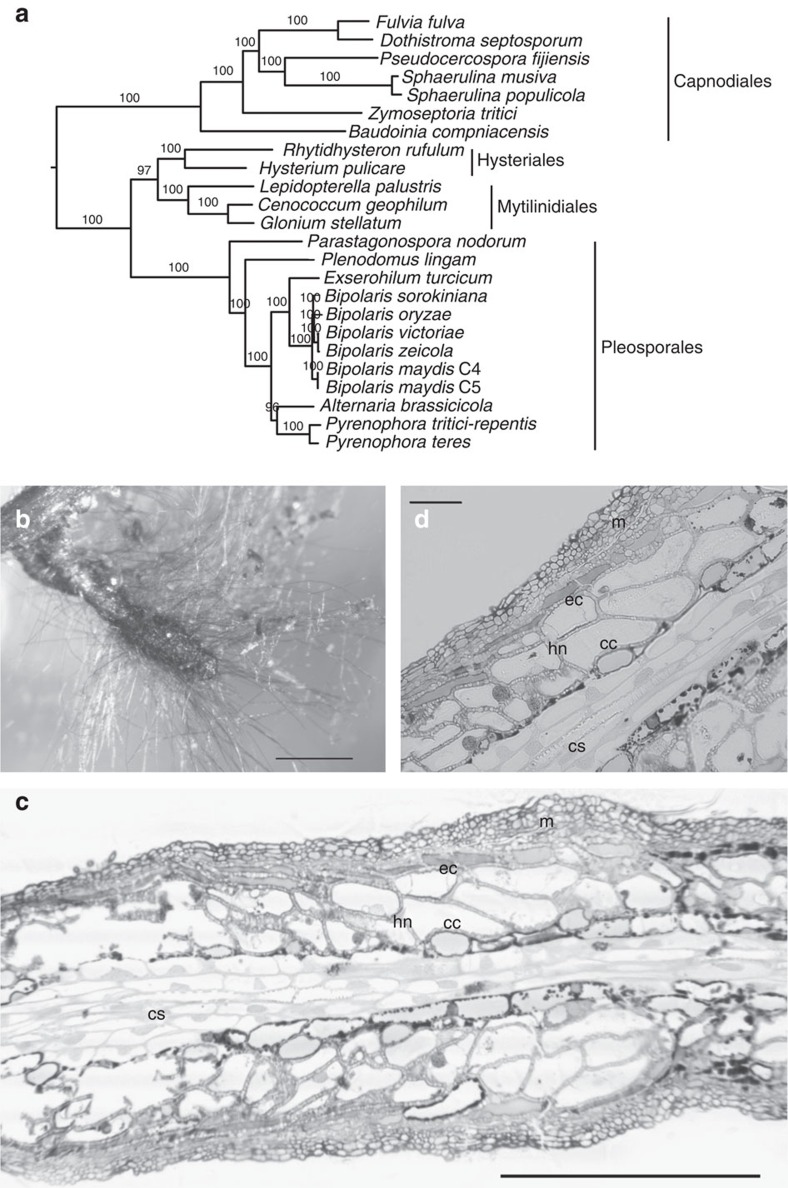
The ectomycorrhizal ascomycete *Cenococcum geophilum*. (**a**) Phylogenomic placement within Dothideomycetes. The tree including all selected species is shown in Supplementary Fig. 1. (**b**) An ectomycorrhizal (ECM) root tip of *C. geophilum-Pinus sylvestris* showing darkly melanized hyphae emanating from the black mantle, and (**c**,**d**) longitudinal sections of a *C. geophilum-P. sylvestris* ECM root tip showing the mantle (m), the Hartig net hyphae (hn) between epidermal (ec) and cortical (cc) root cells as well as the central stele (cs). Scale bars, 1 mm in **b**; 0.2 mm in **c**; 50 μm in **d**.

The genome of *C. geophilum* was assembled to 178 Mb, one of the largest fungal genomes ever sequenced, and is predicted to contain 14,748 gene models (Supplementary Table 1 and JGI MycoCosm portal^14^). The 4-fold smaller 41 Mb and 46 Mb genomes of *Glonium stellatum* and *Lepidopterella palustris* revealed similar gene counts with 14,362 and 13,870 predicted gene models, respectively. Phylogenomic analyses confirmed the placement of *C. geophilum* in Dothideomycetes, order Mytilinidiales, and the close relationship with the saprotrophic *G. stellatum.* (Fig. 1, Supplementary Fig. 1). Intriguingly, *L. palustris*, an aquatic saprotroph, was resolved as closely related to these two species, and thus was included in comparative analyses of Dothideomycetes and other selected species from Ascomycota and Basidiomycota (Supplementary Data 1).

### Repeated elements and gene content

**Figure 2:**
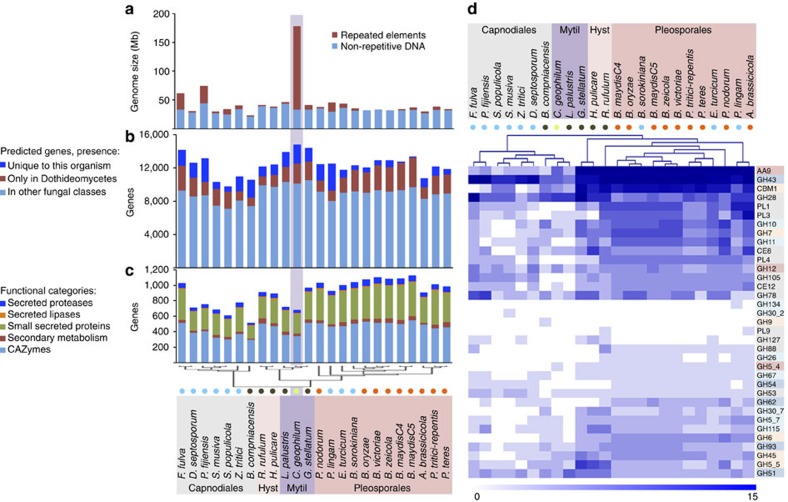
Genome characteristics of *Cenococcum geophilum* as compared with other Dothideomycetes fungi with diverse lifestyles. (**a**) Genome size and repeat element content, (**b**) number of predicted genes broken down by level of conservation, (**c**) number of genes in different selected functional categories. (**a**–**c**) Are grouped according to phylogeny; (**d**) represents a double hierarchical clustering of plant cell wall degrading (PCWD) enzymes, which largely follows the phylogeny of Dothideomycetes but clearly separates the Mytilinidiales according to the reduced numbers of these genes in *C. geophilum* and *L. palustris*. Blue-intensity corresponds to the number of gene counts. PCWDs colour background: Families targeting cellulose (light red), cellulose/hemicellulose (dark red), hemicellulose (light blue), hemicellulose/pectin (dark blue), pectin (grey). Lifestyles: circle colour blue, (hemi)biotrophic plant pathogens; orange, necrotrophic plant pathogens; black, saprotrophs; green, mycorrhiza. For full names of species, see Supplementary Data 1.

The larger genome size of *C. geophilum* compared with its close relatives is explained by its high content (81%) in repeated sequences, mainly consisting of transposable elements (TE; Fig. 2a, Supplementary Data 2). Many plant pathogenic fungi show an increased content in TE, particularly (hemi)biotrophic ones^15^ and this trend is even more striking for symbiotic mycorrhizal fungi (Supplementary Fig. 2).

There is considerably less variation in gene count than genome size, the former ranging from 9,739 to 14,748 in Dothideomycetes with Mytilinidiales at the upper margin (Fig. 2b, Supplementary Data 3). *C. geophilum* has 2,176 unique genes within 1,422 gene families; the largest families, which hold 5 to 30 members (63 families), contain small (<300 amino acids), mostly functionally unknown and often truncated proteins that show no match to the Pfam domain database^16^ (76% of families). Three of them are transposon-related (transposase, helicase), among which the third most-abundant family holding 22 proteins, but they are only weakly expressed, as are most of these large families, with 50% of them showing no expression under any of our experimental conditions (Supplementary Data 4). Similar numbers of unique genes are present in the taxonomically closely related saprotrophic *G. stellatum* and *L. palustris*, the numbers of which range from 48 to 3,152 in Dothideomycetes with fewer unique genes for species with a sequenced close relative^13^ (Fig. 2b, Supplementary Data 3). Expanded gene families in *C. geophilum* are mostly transposon-related or involved in protein–protein interactions and show domains typically found in self/non-self recognition proteins, referred to as somatic incompatibility and defense mechanisms^17^ (for example, HET, NACHT, WD40; Supplementary Data 5). The expression of most of them is not regulated in functioning mycorrhizas.

Although *C. geophilum* is taxonomically more closely related to *G. stellatum* than *L. palustris,* the most recent common ancestors roughly estimated to be 26 and 71 million years ago, respectively (Supplementary Fig. 3), *L. palustris* is more similar to *C. geophilum* with respect to the content in gene categories (Fig. 2c). Although *C. geophilum* and the aquatic *L. palustris* are more similar to the hemiobiotrophic Capnodiales which generally have lower numbers of genes in categories that have been implicated in plant pathogenesis^13^ such as secreted hydrolytic enzymes, the saprotroph *G. stellatum* is more similar to the saprotrophic Hysteriales and necrotrophic Pleosporales (Fig. 2c, Supplementary Data 6). For example, the number of secreted enzymes of *C. geophilum, L. palustris* and *G. stellatum* are 595, 588 and 823, respectively, whereas sequenced Capnodiales, Hysteriales and Pleosporales have on average 629, 830 and 894 of these genes in their gene repertoire.

### Plant cell wall degrading enzymes and secondary metabolism

The observed division of Mytilinidiales based on the species' gene contents is most notable for PCWDEs where double hierarchical clustering largely follows the phylogeny of Dothideomycetes but clearly separates Mytilinidiales according to the reduced numbers of these genes in *C. geophilum* and *L. palustris* (Fig. 2d). This reduction in gene content can also be noted for enzymes acting on cellulose, hemicellulose and pectin (Supplementary Data 7). The lytic polysaccharide monooxygenases (LPMO; AA9) for example acting on cellulose and hemicellulose are significantly (Fisher's exact test *P*=0.001) reduced to 5, 2 and on average two members in *C. geophilum*, *L. palustris* and Capnodiales compared with 17 in *G. stellatum* and on average 25 in the Hysteriales/Pleosporales, respectively (Supplementary Data 7). Likewise, as noted previously for the white-rot versus brown-rot modes of wood decay^18^ and for ECM basidiomycetes^1^, enzymes degrading crystalline cellulose (for example, cellobiohydrolases GH6 and GH7) or modules binding (for example, CBM1) are significantly reduced from 3, 5 and 10 members on average for *G. stellatum*/Hysteriales/Pleosporales to 0, 1 and 2 members for *C. geophilum*/*L. palustris*/Capnodiales, respectively. Xylanases (GH10, GH11), mannanases (GH26) and glucuronidases (GH115) acting on hemicellulose as well as pectin attacking enzymes (PL1, PL3, PL4, CE12) are also reduced from two to five members to no or only one member.

**Figure 3:**
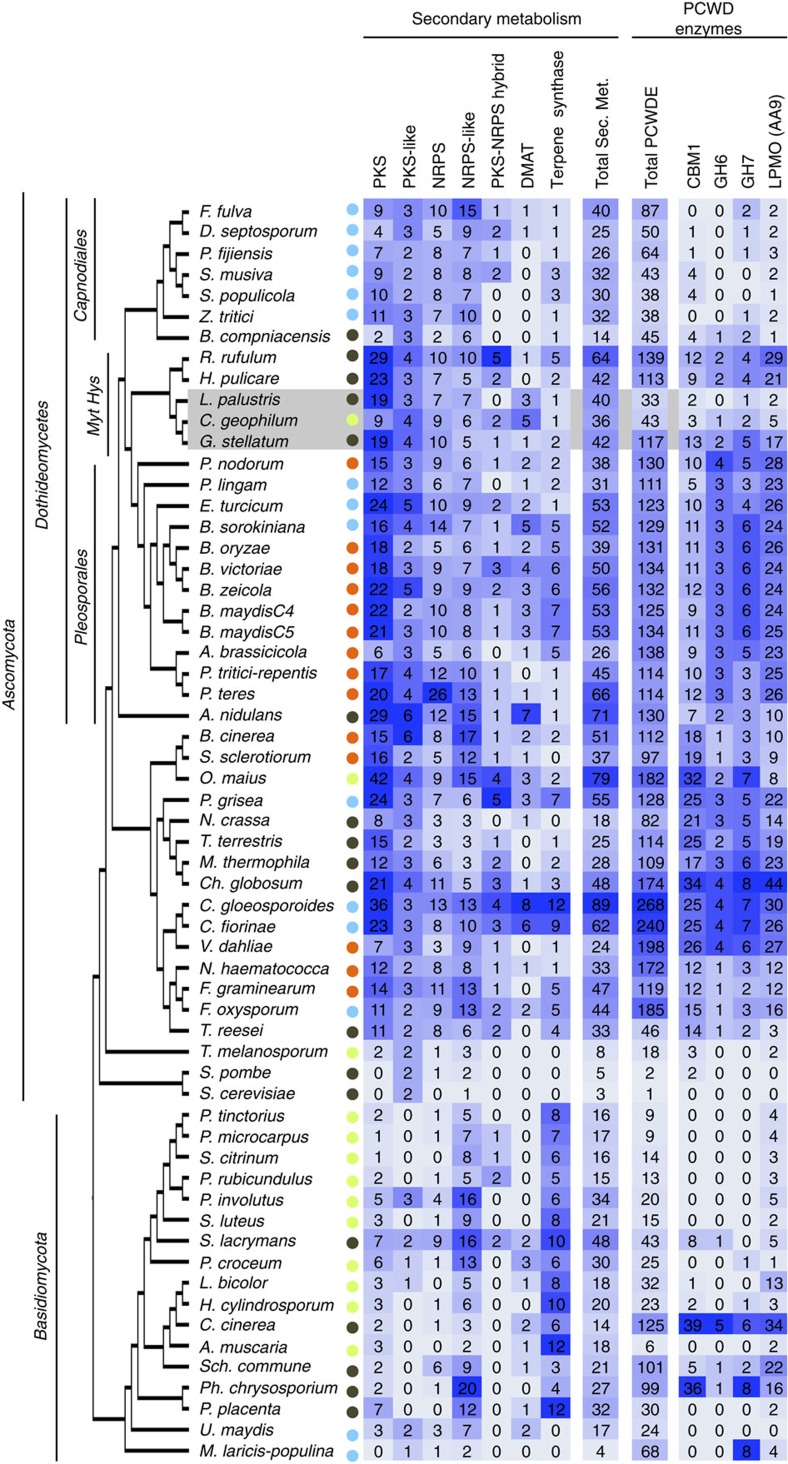
Genes coding for secondary metabolism and selected plant cell wall degrading enzymes in fungi with diverse lifestyles. Circle colour blue, (hemi)biotrophic plant pathogens; orange, necrotrophic plant pathogens; black, saprotrophs; green, ectomycorrhizal and ericoid mycorrhizal (*O. maius*) fungi. PCWD enzymes, plant cell wall-degrading enzymes; PKS, polyketide synthase; DMATS, prenyltransferase; NRPS, nonribosomal peptide synthase. The heat map depicts gene numbers shaded in bright to dark blue according to their number. For full names of species, see Supplementary Data 1.

Reduced decay capabilities of plant cell walls are a hallmark of the ECM symbionts in Agaricomycotina^1^ and Tuberaceae^2^, a lifestyle feature, which also convergently evolved in Dothideomycetes (Fig. 2d, Supplementary Data 7 and 8). Generally, ECM fungi show significantly smaller numbers and less variation of total PCWDEs than saprotrophic and pathogenic fungi (Supplementary Fig. 4). Total numbers range between 6 and 43 with a mean of 19 PCWDEs in ECM fungi, whereas necrotrophic plant pathogens have on average 132 PCWDEs (Supplementary Data 7). Although the total numbers of PCWDE is lower within ECM fungi with even missing families (for example, cellobiohydrolases GH6 and GH7), ECM fungi retained a limited and unique set of these enzymes including several polysaccharide lyases (GH28; ref. 1). Of all ECM fungi sequenced so far, *C. geophilum* shows the highest PCWDE (43 enzymes) and SM repertoires (Fig. 3). For example, the proteins targeting crystalline cellulose (GH6, GH7, AA9, CBM1) are present in the *C. geophilum* genome (Fig. 3), whereas they are often absent in other ECM fungi^1^. The number of SM genes, many members of which are known to be involved in pathogenic interactions^13^,^19^, is not reduced in the genome of *C. geophilum* except for polyketide synthases; SM genes are generally more numerous than in ECM basidiomycetes^13^,^20^ (36 SM genes in *C. geophilum* compared 23 SM genes on average for ECM basidiomycetes; Fig. 3). However, most retained PCWDEs are only weakly expressed in ECM root tips (for example, GH12, GH5_5, GH5_7, GH51, CE8; Supplementary Fig. 5) and are potentially involved in the colonization of the root apoplastic space during Hartig net formation. The expression of most genes related to SM are downregulated in ECM, except for two nonribosomal peptide synthases (NRPS, Cenge3:701851 and Cenge3:471114) and a PKS (Cenge3:706069; Supplementary Data 9). Interestingly, the former NRPS shows a high protein sequence identity (42%) to *Pes1* of *Aspergillus fumigatus* that confers protection against oxidative stress^21^, which is an inevitable consequence of drought but also imposed by plants as a defense mechanism against biotic stresses^22^.

### Mycorrhiza-induced small secreted proteins

**Figure 4:**
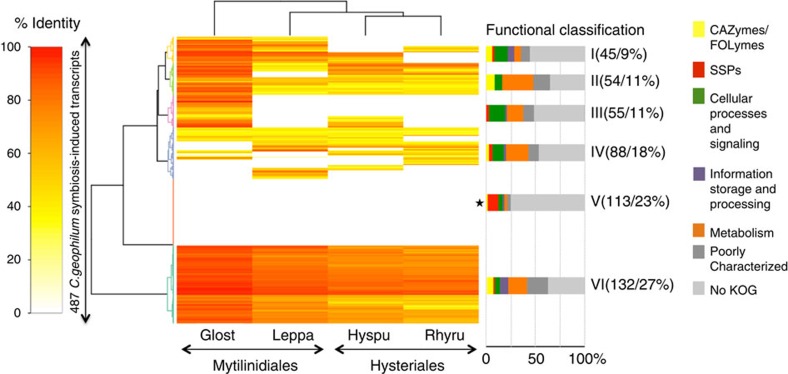
Sequence conservation and functional analysis of symbiosis-induced transcripts. The heatmap depicts a double hierarchical clustering of 487 symbiosis-upregulated *Cenococcum geophilum* genes (rows, fold change >5, FDR-corrected *P*<0.05). Symbiosis-induced genes were blasted (BLASTP) against the genomes of *Glonium stellatum* (Glost), *Lepidopterella palustris* (Leppa), *Hysterium pulicare* (Hyspu) and *Rhytidhysteron rufulum* (Rhyru) to find homologues (see Supplementary Fig. 6a and b for a comparison with 50 fungal genomes and Supplementary Fig. 6c and d for downregulated genes). Homologues are coloured from yellow to red depending on the percentage of similarity. Left of heatmap, clusters are highlighted by branches of the same colour. Right of heatmap, the percentages of putative functional categories are given for each cluster as bargrams and the number and percentage of genes in each cluster are shown. Clusters significantly enriched in small secreted proteins (SSPs) are marked with an asterisk (Fisher's exact test *P*<0.01). CAZyme, carbohydrate active enzymes; FOLyme, fungal oxidative lignin-degrading enzymes; no KOG, nonsecreted orphan genes.

By comparing sequenced RNA from mycorrhizal roots and free-living mycelia, we identified 3% of the genes being upregulated in symbiosis (fold change >5, false discovery rate-corrected *P* value <0.05; Supplementary Data 10). They are classified into similar functional categories depending on the grade of conservation as seen for other mycorrhizal fungi^1^ involved in signalling, information storage and processing, and metabolism (Fig. 4, Supplementary Fig. 6a and b; downregulated genes in Supplementary Fig. 6c and d). Among the most highly expressed and upregulated genes in symbiosis are transporters and small proteins of less than 300 amino acid residues with a predicted signal peptide (small secreted proteins; SSPs; Table 1), the latter significantly being enriched in the symbiosis-induced genes compared with the fraction present in the genome (4% compared with 2% in the whole genome, *P*=6.60 × 10^−5^, Fisher's exact test; Supplementary Fig. 6b). Eighteen percent (by comparing with 50 genomes; Cluster VI in Supplementary Fig. 6a and b) to 23 per cent (by comparing with the closely related Mytilinidiales and Hysteriales genomes, Cluster V in Fig. 4) of the upregulated genes are taxon-specific since they are only found in *C. geophilum*, and SSPs are again overrepresented in these taxon-specific orphan genes compared with the percentage in the total gene repertoire (10% versus 2%; *P* value <0.01, Fisher's exact test). These SSPs might encode new symbiosis-related effectors, as observed in *Laccaria bicolor* where the mycorrhiza-induced protein MiSSP7 controls the defense-related jasmonate pathway in host roots^1^,^3^.

### Aquaporins

**Figure 5:**
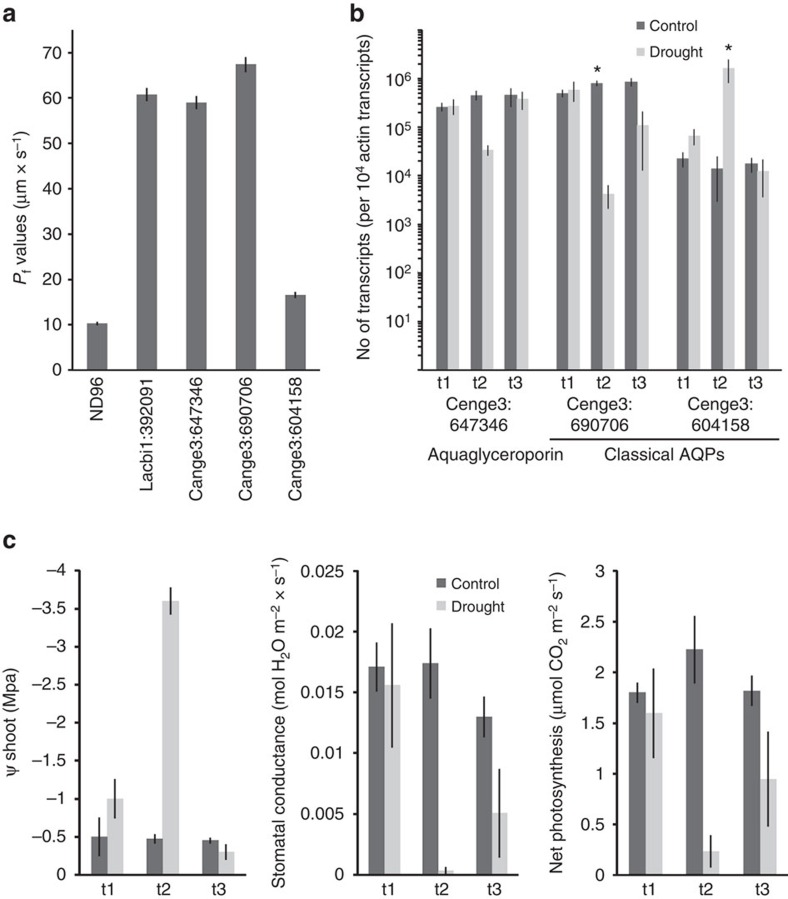
Functional characterization of three selected aquaporins of *Cenococcum geophilum*. Two aquaporins (AQP) were highly symbiosis-induced (Cenge3:647346, Cenge3:690706),whereas Cenge3:604158 was not regulated by the symbiosis. (**a**) Water permeability of *Xenopus laevis* oocytes expressing *C. geophilum* AQPs as compared with ND96-buffer injected oocytes (negative control) or oocytes expressing a *Laccaria bicolor* AQP (positive control; mean *P*_f_ values, *n*=20±s.e.m.). Compared with the ND96-buffer injected control, all three *C. geophilum* AQPs significantly increased the plasma membrane water permeability of oocytes (*P* values of Student's *t*-test: 1.5E−51, 4.7E−55, 1.6E−5, respectively). (**b**,**c**) Impact of drought and rewatering on the fungal AQP expression in mycorrhizal root tips of Scots pine (*Pinus sylvestris*)/*C. geophilum* seedlings grown in pots in a climate chamber experiment. (**a**) Gene expression of the three AQPs at three time points (t1, 10 days of drought; t2, 14 days of drought; t3, 3 days after rewatering) in ECM root tips of well-watered (control) and drought-stressed pine seedlings (means of four replicates (pots) ±s.e.m.). Asterisks indicate significance of the drought treatment (*P*<0.05, analysis of variance). (**c**) Shoot water potential, stomatal conductance and net photosynthesis of the Scots pine seedlings measured at the three time points (means of four replicates (pots) ±s.e.m.).

Intriguingly, two of the three most highly upregulated and expressed genes in ECM are coding for aquaporins (AQP) that channel water and/or small solutes across membranes^23^ (Table 1). This high upregulation seems to be a feature of the *C. geophilum* symbiotic interaction when compared with other mycorrhizal fungi (Supplementary Fig. 7), although important functions of fungal AQPs in the ECM symbiosis have been shown for other ECM fungi^24^,^25^,^26^. *C. geophilum* harbours six putative functional aquaporins in its genome, a similar number to closely related Dothideomycetes relatives (Supplementary Data 11). Four are classified as classical AQPs, which facilitate mainly water transport, one aquaglyceroporin that may also channel small solutes such as urea, glycerol and ammonia, and one X intrinsic protein of which the function is less clear^23^ (Supplementary Fig. 8). To assess the role of the two symbiosis-induced AQPs (the aquaglyceroporin Cenge3:647346 and a classical AQP Cenge3:690706) as well as an additional classical AQP (Cenge3:604158), we studied both their water transport capabilities and expression patterns in ECM subjected to severe drought and rewatering and in the free-living mycelium under drought. The two symbiosis-upregulated AQPs enabled similar high water permeability of *Xenopus laevis* oocyte plasma membranes, whereas permeability of Cenge3:604158 was low but still significantly increased compared with buffer-injected controls (Fig. 5a). Interestingly, the two symbiosis-induced AQPs (Cenge3:647346 and Cenge3:690706) were downregulated, whereas the additional classical AQP (Cenge3:604158) was highly upregulated upon high drought stress in ECM of Scots pine (*Pinus sylvestris*) seedlings (Fig. 5b,c) and in free-living mycelia (Supplementary Fig. 9, statistics in Supplementary Table 2). We followed plant physiological parameters over the drought/rewatering experiment to assess the intensity of the drought treatment (Fig. 5c). After 9 days without watering, the seedlings hardly experienced drought yet (mean shoot water potential, −1 MPa) neither significantly affecting the plants gas exchange nor fungal AQP expression. At high drought stress (shoot water potential of −3.5 MPa), gas exchange ceased in most plants and they started to recover 2 days after rewatering. Similarly, a strong downregulation of the two symbiosis-induced fungal AQPs was observed at high drought stress, whereas the expression of the classical AQP Cenge3:604158 increased upon drought, reaching the highest transcript level of all *C. geophilum* AQPs (Fig. 5b,c). Transcript levels of the three AQPs returned completely (Cenge3:647346, Cenge3:604158) or almost (Cenge3:690706) to the controlled state 2 days after rewatering. Interestingly, under well-watered control conditions, the transcript levels of the drought-induced, classical AQP (Cenge3:604158) correlated best with the shoot water potential of their host plant (Pearson correlation 0.70), whereas the symbiosis-induced classical AQP significantly positively correlated with the nitrogen content of the needles (0.62) and the plant's water use efficiency (0.60; Supplementary Fig. 10).

In the same drought/rewatering experiment, we assessed the plant conditions of mycorrhizal versus non-mycorrhizal (NM) plants. We observed significantly increased needle nitrogen content, net photosynthesis and water use efficiencies in ECM compared with NM plants, confirming the mutualistic fungus–plant relation. On the other hand, no significant effect of mycorrhizal inoculation was observed in the drought treatment (Supplementary Fig. 11, Supplementary Table 3).

### Symbiosis-regulated plant genes

**Figure 6:**
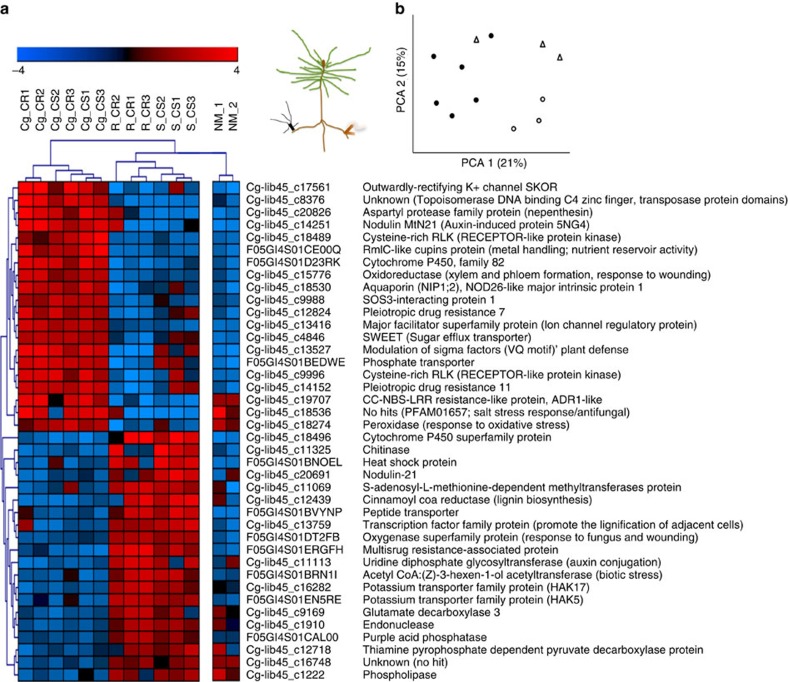
Scots pine differentially interacts with *Cenococcum geophilum* and other simultaneously inoculated mycorrhizal fungi. (**a**) The 20 most highly up- or downregulated Scots pine (*Pinus sylvestris*) genes expressed in ectomycorrhizas (ECM) of double-inoculated seedlings (CR, *C. geophilum* and *Rhizopogon roseolus*; CS, *C. geophilum* and *Suillus granulatus*; three replicate plants each) when interacting with C*. geophilum* (Cg) compared with *S. granulatus* (S) or *R. roseolus* (R). Expression of genes in non-mycorrhizal plants (NM; two replicate plants) is also indicated. The heat map depicts a double hierarchical clustering in which normalized gene expression values are coloured from red (up-) over black (mean expression over all conditions) to blue (downregulated). (**b**) Principal component analysis (PCA) based on the expression of 7,000 *P. sylvestris* genes shows that *C. geophilum* interacting ECM root tips (filled circles) are separated on the first axis from the other two ECM fungi (*R. roseolus*, open triangles and *S. granulatus*, open circles). Each symbol per fungal species corresponds to a plant replicate. Note the sketch showing a pine seedling colonized by *C. geophilum* (left root) and one of the other fungi (right root).

To identify specific gene networks induced in host roots by *C. geophilum*, we inoculated seedlings of Scots pine simultaneously with *C. geophilum* and either *Suillus granulatus* or *Rhizopogon roseolus*, two common ECM fungi associated to pines^27^. We then measured the differential expression of Scots pine genes in the respective mycorrhizas using oligoarrays. Although 8% of these genes were differentially regulated in mycorrhizal compared with NM roots (*P*<0.05; posterior probability of differential expression^28^ PPDE>0.97, Supplementary Data 12), the expression of 1% significantly differed in ECM root tips challenged by *C. geophilum* compared with the other two symbionts (Fig. 6 and Supplementary Data 13). Several interesting patterns emerged from these data: (1) Genes for membrane transporters, such as a the SWEET sugar exporters (11.1 × fold change in *C. geophilum* interacting ECM tips compared with NM tips versus 4.2 × and 1.7 × in *S. granulatus* and *R. roseolus* ECM tips compared with NM tips, respectively; 3.7 × fold change *C. geophilum* versus other symbionts, PPDE 0.99), ammonium and phosphate transporters (1.6–5.2 × fold change in *C. geophilum* ECM versus other symbionts) and a NOD26 AQP (5.6 × fold change in *C. geophilum* ECM versus other symbionts, PPDE 0.99) were more highly upregulated in *C. geophilum* ECM than in ECM root tips of the other two fungi possibly indicating a higher nutrient and water transfer. (2) A K^+^ efflux transporter SKOR, which is induced by stressors and reactive oxygen species mediating K^+^ export in xylem vessels and which is thought to be involved in stress signalling^29^, was expressed 18.4-fold higher (PPDE 0.98) in ECM of *C. geophilum* than in the other ECM fungi. (3) Many oxidative stress- and pathogen resistance-related proteins such as peroxidases, anthocyanin biosynthesis^30^ and SOS interacting proteins^31^ were upregulated in *C. geophilum* ECM compared with NM root tips and downregulated in roots tips interacting with other ECM fungi (3.5–5.6 × fold change in *C. geophilum* versus other symbionts). Most remarkably, the gene showing the second highest differential expression (20.4 × fold change, PPDE 1.00) codes for an activated disease resistance (ADR1)-like protein, which is usually induced by plant pathogens and conveys broad-spectrum disease resistance^32^. Enhanced expression of this gene leads to drought tolerance in *Arabidopsis,* which suggests that interlinked networks of abiotic and biotic stress signalling exist and may have significant functional overlap^32^.

### Presence of sex-related genes in *C. geophilum*

A puzzling finding of ECM field surveys is the observed global distribution and high genetic diversity of *C. geophilum*, a presumably asexual soil fungus relying on vegetative mycelial propagation^9^,^10^. We therefore assessed whether *C. geophilum* has the prerequisites to have sex and to produce fruiting bodies. The genome of *C. geophilum* strain 1.58 contains a *MAT* locus, which is conserved with its close relative *G. stellatum* (Supplementary Fig. 12). It includes one intact mating-type gene (*MAT1-1-1*) of the two typically present in the bipolar mating system of ascomycetes (*MAT1-1-1* and *MAT1-2-1*), indicating that *C. geophilum* is self-sterile^33^. Genes encoding proteins of the pheromone response pathway and involved in fruiting body production are equally present in *C. geophilum* and *G. stellatum* (Supplementary Data 14), indicating that *C. geophilum* is possibly able to reproduce sexually by outcrossing and to differentiate inconspicuous fruiting bodies, maybe similar to those produced by *G. stellatum*^12^.

## Discussion

The *C. geophilum* genome contains many features of the symbiosis molecular toolbox found in the ECM fungi sequenced so far, that is, loss of genes involved in PCW degradation and presence of a large set of taxon-specific, symbiosis-upregulated orphan genes, including MiSSPs^1^. These findings support the independent convergent evolution of this ecology in Dothideomycetes, which diverged from other fungal classes approximately 280 million years ago^12^,^13^ and which is the largest class of ascomycetes. *C. geophilum* is the only known ECM member in this class comprising over 19,000 species with an unparalleled diversity of lifestyles^13^ and including many devastating plant pathogens. This prompted the genome sequencing of >100 Dothideomycetes providing an excellent opportunity for comparative genomics to identify the peculiarities of the ECM lifestyle.

One of the most obvious and outstanding features of the *C. geophilum* genome is its very large size resulting from ancient TE bursts (Fig. 2). Mycorrhizal fungi, such as the ECM *Tuber melanosporum* and the arbuscular mycorrhizal *Rhizophagus irregularis*, generally show an increased proportion of TE, as do many biotrophic pathogens^34^. However, several ECM fungi, such as *Hebeloma cylindrosposrum* and *Amanita muscaria* (Supplementary Data 2) did not experience massive TE proliferations, suggesting this is not a prerequisite of the biotrophic lifestyle. Genome invasion by TEs might explain the success of *C. geophilum* to colonize a wide range of hosts and habitats^5^ since TE are known to contribute to the plasticity and adaptability of fungi to their environment^15^,^35^,^36^. The high content in TE might partly explain the high genetic diversity and cryptic speciation observed in *C. geophilum* populations^9^,^10^,^11^. On-going genome re-sequencing of geographic isolates of *C. geophilum* should provide additional information about the role of TE in genetic structuration of the symbiont's populations.

There is considerably less variation in gene count than genome size in Dothideomycetes as previously noted^13^ (Fig. 2). However, gene content varies widely, in particular in categories found to be involved in pathogenesis such as those coding for plant-cell-wall degrading and secondary metabolism enzymes^37^. Most remarkably, this can be noted within Mytilinidiales, where a reduced number of PCWDE separates *C. geophilum* and the aquatic saprotroph *L. palustris* from the saprotroph *G. stellatum* (Fig. 2), the latter likely showing the ancestral state of this family placed within Pleosporomycetidae^38^. A reduced complement of such genes is meaningful for (hemi)biotrophs to avoid triggering plant defense mechanisms, one of the suggested evolutionary adaptations to the biotrophic lifestyle^13^,^34^ in both, pathogenic and mutualistic interactions^39^. Although ECM fungi show consistently reduced numbers of PCWDEs, (hemi)biotrophic pathogens are more variable (Supplementary Fig. 4), which might depend on their strategy to enter plant tissues and cells, evade plant defense mechanisms and switch between biotrophic and necrotrophic phases. Saprotrophs benefit from a large repertoire of PCWDEs to efficiently acquire nutrients from the environment, but here also a wide range of lignocellulose plant decay capabilities is evident as revealed by the contrasted gene repertoires of white- and brown-rot decayers^18^. Whether the reduction in PCWDE observed for *L. palustris* is typical for aquatic saprotrophs is doubtful and might rather be an adaptation of this species to its ecological niche within freshwater fungal communities since diverse and high degradative abilities have been documented for other aquatic fungi^40^. Yet, genome resources are scarce to confirm this.

Having arisen from functionally diverse wood- and litter-decay saprotrophs, ECM fungi retained distinct arrays of PCWDEs^1^,^2^,^41^ and since residing within Pleosporomycetidae^38^, the rather high fraction of such genes in *C. geophilum* does not come as a surprise. Those expressed in ECM roots (Supplementary Fig. 5) are likely involved in the deconstruction of the middle lamella accompanying the formation of the Hartig net. However, most of the retained PCWDEs are not expressed in ECM, neither are the genes related to secondary (toxin) metabolism. They therefore likely play a role in developmental stages other than those involved in the mutualistic fungus-plant interaction in vital and functioning ECM. For example, they might participate at later ECM stages in root turnover after senescence. Still, the increased expression of stress- and pathogen resistance-related proteins (for example, receptor-like protein kinase, NBS-LRR disease proteins) seen in the plant hosts transcriptome when associated with *C. geophilum* compared with the ECM basidiomycetes *S. granulatus* and *R. roseolus* (Fig. 6, Supplementary Data 13) indicate that these pathogenic-related features might affect the mutualistic interaction to some extent. The most highly regulated plant gene with a known function, the activated disease resistance gene (*ADR1*), increases drought resistance in *Arabidopsis thaliana* possibly by linking signalling networks of biotic and abiotic stress^32^. We therefore speculate that *C. geophilum* colonization primes pathways conferring drought tolerance as suggested previously for other organisms^42^.

The striking upregulation of genes coding for taxon-specific SSPs in symbiosis confirms previous observations^1^,^41^ and highlights the importance of these proteins in mutualistic interactions. The role of such candidate effector proteins has been extensively reported in pathogenic systems^34^,^43^ and they have recently been characterized in mycorrhizal mutualists^3^,^44^. Besides the MiSSP7 effector secreted by the ECM *Laccaria bicolor*, the key role of secreted effectors of the arbuscular mycorrhizal fungus *Rhizophagus irregularis* and the beneficial endophyte *Piriformospora indica* in subverting plant immunity has been confirmed^44^, but many MiSSPs await further characterization. Analysis of the role of *C. geophilum* MiSSPs in the symbiotic interaction is currently underway. Because of the low protein sequence conservation of secreted effector-like proteins, their function cannot be inferred by comparisons with known genes of taxonomically related pathogenic relatives. There are indications that in spite of sequence divergence, functional redundancy of secreted effectors exists even between pathogenic and mutualistic fungi, with conserved targets in their plant hosts (for example, JAZ repressors implicated in defense-related pathways)^44^,^45^. If this holds true for other mutualistic effector proteins, the question will be: what determines the outcome of their activity, beneficial or pathogenic, depending on the fungal lineage? For ECM interactions, *C. geophilum* might serve as a favourable model system for such studies because knowledge of several well-studied pathogenic relatives is already available^13^,^37^.

Gene expression profiling revealed changes in the expression of AQP genes coding for water channels in symbiosis. The high expression of two highly water permeable AQPs (Cenge3:647346 and Cenge3:690706) in functioning ECM may be provoked by plant water and/or nutrient demand upon the interaction. Expression analyses indicate that a fine-tuned regulation of the AQP genes occurs under drought conditions. However, in our experiment, we could not show a significant effect of *C. geophilum* mycorrhization on plant physiological parameters under drought and therefore more studies in this regard are needed.

Owing to its worldwide distribution, general mutualistic behaviour, high genetic polymorphism and its possible role in host plant water and nutrient metabolism, *C. geophilum* population genomics should shed light on the mechanisms of host and environmental adaptation. It should facilitate the identification of drought-adapted *C. geophilum* strains, which can be used to efficiently support their host trees threatened by the forecasted increase in drought periods in many parts of the world^46^.

## Methods

### Fungal strains used for genome sequencing

*Cenococcum geophilum* Fr. strain 1.58 was isolated from a sclerotium in April 2008. It was collected in an irrigation experimental site in the Pfyn forest, which is dominated by Scots pines and situated in a dry inner alpine valley in Switzerland^47^. Strains from *Glonium stellatum* Muhl. and *Lepidopterella palustris* Shearer & J.L. Cranes were ordered from CBS Fungal Biodiversity Centre (NL). *Glonium stellatum* strain CBS207.34 was isolated in Michigan (USA) from oak and deposited in 1934. *Lepidopterella palustris* strain 459.81 was isolated from a submerged twig in a cypress swamp located in Johnson County, Illinois (Deer Pond) in 1977 and deposited at CBS in 1981.

### Genome sequencing and assembly

For genomic DNA sequencing, mycelia of *C. geophilum and G. stellatum* were grown in liquid culture containing Cg-Medium, a modified MMN medium containing casein as outlined in Kerner *et al*.^48^ or on potato dextrose broth for *G. stellatum*. Three to 8 weeks after inoculation, the mycelia were harvested, pulverized in liquid nitrogen and stored at −80 °C until processing. DNA was extracted using either the PowerMax Soil DNA isolation kit (MOBIO) according to the manufacturer's instructions, the Qiagen DNeasy plant Mini kit (*G. stellatum*) or using the protocol outlined by Kohler *et al*.^1^. *Lepidopterella palustris* mycelium was grown in liquid culture containing malt extract medium (20 g malt extract, 0.5 g yeast extract per litre) for 1 to 2 weeks before DNA was extracted using the Qiagen (Valencia, CA) Genomic 500/G tips. *C. geophilum* and *L. palustris* were sequenced at the JGI facilities, while *G. stellatum* was sequenced within the AFTOL project (aftol.org). For the *C. geophilum* genome sequencing, two Illumina fragment and three long mate pair (LMP) libraries have been sequenced, assembled with AllPathsLG^49^, and further improved with Pacific Biosciences sequencing as follows: For fragment libraries, 500 ng of genomic DNA was sheared using the Covaris E210 (Covaris) and size-selected using Agencourt Ampure Beads (Beckman Coulter). The DNA fragments were treated with end repair, A-tailing and adapter ligation using the TruSeq DNA Sample Prep Kit (Illumina) and purified using Agencourt Ampure Beads (Beckman Coulter). Two types of LMP libraries were prepared. LFPE (ligation-free paired end) mate pair fragments were generated using the 5500 SOLiD Mate-Paired Library Construction Kit (SOLiD). Fifteen micrograms of genomic DNA was sheared using the Covaris g-TUBETM (Covaris) and gel size selected for 4–4.5 kb. The sheared DNA was end repaired and ligated with biotinylated internal linkers. The DNA was circularized using intramolecular hybridization of the internal linkers. The circularized DNA was treated with plasmid safe to remove non-circularized products. The circularized DNA was nick translated and treated with T7 exonuclease and S1 nuclease to generate fragments containing internal linkers with genomic tags on each end. The mate pair fragments were A-tailed and purified using Strepavidin bead selection (Invitrogen). The purified fragments were ligated with Illumina adaptors and amplified using 10 cycles of PCR with Illumina primers (Illumina) to generate the final library. In addition, CreLox library was produced from 15 μg of genomic DNA, which was sheared using the Hydroshear and gel-size selected for 4.5 kb. The fragments were end-repaired (NEB) and ligated with biotinylated Illumina compatible adapters containing loxP (IDT). The adapter ligated DNA fragments were circularized via recombination by a Cre excision reaction (NEB). The circularized DNA was digested using four base cutter restriction enzymes followed by self-ligation (NEB). The self-ligated products were immobilized on beads and inverse PCR was used to enrich for the final library (NEB). Both the libraries were quantified using KAPA Biosystem's next-generation sequencing library quantitative PCR (qPCR) kit and run on a Roche LightCycler 480 real-time PCR instrument. The quantified libraries were then prepared for sequencing on the Illumina HiSeq sequencing platform utilizing a TruSeq paired-end cluster kit, v3, and Illumina's cBot instrument to generate a clustered flowcell for sequencing. Sequencing of the flowcell was performed on the Illumina HiSeq2000 sequencer using a TruSeq SBS sequencing kit 200 cycles, v3, following a 2 × 100 and 2 × 150 indexed run recipe for LFPE and fragments, respectively. Genomic data from five libraries were filtered and assembled with AllPathsLG^49^. To fill gaps in Illumina genome assembly of *C. geophilum*, an unamplified library was generated using Pacific Biosciences standard template preparation protocol, where 5 μg of gDNA was used to generate the library and the DNA was sheared using a Covaris LE220 focused-ultrasonicator with their Blue 3.0 kb miniTUBES to generate sheared fragments of 3 kb in length. The sheared DNA fragments were then prepared according to the Pacific Biosciences protocol and using their SMRTbell template preparation kit, where the fragments were treated with DNA damage repair, had their ends repaired so that they were blunt-ended, and 5′ phosphorylated. Pacific Biosciences hairpin adaptors were then ligated to the fragments to create the SMRTbell template for sequencing. The SMRTbell templates were then purified using exonuclease treatments and size-selected using AMPure PB beads. Sequencing primer was then annealed to the SMRTbell templates and Version C2 sequencing polymerase was bound to them. The prepared SMRTbell template libraries were then sequenced on a Pacific Biosciences RS sequencer using Version C2 chemistry and running 2 × 45 min movies per SMRT cell.

*Lepidopterella palustris* was sequenced at JGI using one fragment and one LMP libraries. For the fragment library, 100 ng of DNA was sheared to 270 bp using the covaris LE220 (Covaris) and size selected using SPRI beads (Beckman Coulter). The fragments were treated with end-repair, A-tailing and ligation of Illumina compatible adaptors (IDT, Inc) using the KAPA-Illumina library creation kit (KAPA Biosystems). In addition, LFPE (ligation-free paired end) mate pair fragments were generated using the 5500 SOLiD Mate-Paired Library Construction Kit (SOLiD) as described for *C. geophilum*. The purified fragments were ligated with Illumina adaptors and amplified using eight cycles of PCR with Illumina primers (Illumina) to generate the final library. Quantification and sequencing were performed as described above for *C. geophilum*. Genomic data from the two libraries were filtered and assembled with AllPathsLG^49^.

*Glonium stellatum* genome sequencing and assembly was performed within the AFTOL project using a single short insert library. For this library, 1.0 μg of genomic DNA was sheared using the Bioruptor XL (Diagenode) and size-selected for 400 bp through agarose gel isolation. The fragments were treated with end-repair, A-tailing and ligation of Illumina compatible adaptors (IDT, Inc) using the NEBNext kits (New England BioLabs). The purified fragments were ligated with Illumina adaptors and amplified using 16 cycles of PCR with Illumina primers (Illumina) to generate the final library. The library was quantified on an Agilent 2100 Bioanalyzer (Agilent) and prepared for sequencing on the Illumina HiSeq sequencing platform utilizing a TruSeq paired-end cluster kit, v3. The library was sequenced one lane of a flowcell on an Illumina HiSeq2000 sequencer using a TruSeq SBS sequencing kit 100 cycles, v3, at the Core Facilities of the Center for Genome Research and Biocomputing at Oregon State University. *De novo* genome assembly of filtered Illumina sequences was performed using VELVET^50^ 1.0, with a minimum contig length of 300 bp. For genome annotations, RNA of mycelia grown in liquid culture with different growth conditions as well as from different tissues for *C. geophilum* (sclerotia and mycorrhizas) were sequenced after total RNA extraction (RNeasy Plant Mini Kit of Qiagen with integrated DNase step) using the Illumina platform at the JGI facilities (*C. geophilum* and *L. palustris*) and at Beckman Coulter Genomics facilities (*G. stellatum*). *C. geophilum* messenger (mRNA) was purified from total RNA using Dynabeads mRNA Purification Kit (Invitrogen) and chemically fragmented to 200–250 bp (Ambion). mRNA was reverse transcribed with SuperScript II using random hexamers. Second-strand complementary DNA (cDNA) was synthesized using dNTP/dUTP mix (Thermo Scientific), *Escherichia coli* DNA Ligase, *E. coli* DNA polymerase I, and *E. coli* RnaseH (Invitrogen). The fragmented cDNA was treated with end-pair, A-tailing, adapter ligation using the TruSeq Sample Preparation Kit (Illumina). Second-strand cDNA was removed by AmpErase UNG (Applied Biosystems) to generate strandedness.

Stranded cDNA libraries for *L. palustris* were generated using the Illumina Truseq Stranded RNA LT kit. mRNA was purified from 1 μg of total RNA using magnetic beads containing poly-T oligos. The mRNA was fragmented using divalent cations and high temperature. The fragmented RNA was reversed transcribed using random hexamers and SSII (Invitrogen) followed by second-strand synthesis. The fragmented cDNA was treated with end-pair, A-tailing, adaptor ligation and 10 cycles of PCR. Both the libraries were quantified using KAPA Biosystem's next-generation sequencing library qPCR kit and run on a Roche LightCycler 480 real-time PCR instrument. These were prepared for sequencing on the Illumina HiSeq sequencing platform utilizing a TruSeq paired-end cluster kit, v1 and v3 for *L. palustris* and *C. geophilum*, respectively, and Illumina's cBot instrument to generate a clustered flowcell for sequencing. Sequencing of the flowcell was performed on the Illumina HiSeq2000 sequencer using a TruSeq SBS sequencing kit 200 cycles, v1 following a 2 × 100 indexed run recipe for *L. palustris* and v3 with 2 × 150 bp reads for *C. geophilum*. For *G. stellatum,* library construction and 2 × 100 bp Illumina HiSeq sequencing was performed by Beckman Coulter Genomics (Danvers, MA, USA) using their standard protocols.

RNA-Seq reads were assembled using Rnnotator v.2.5.6 or later^51^ or CLC Genomics Workbench v6 or later (Qiagen) after trimming and filtering for low-quality, low-complexity, adaptor and duplications. Assembled contigs of at least 100 bp length with at least three reads mapped were corrected for misassemblies, polished, clustered into contigs to produce final non-redundant virtual transcripts. The genome assemblies of the three fungi were each annotated using the JGI Annotation Pipeline, which detects and masks repeats and transposable elements, predicts genes, characterizes each conceptually translated protein, chooses a best gene model at each locus to provide a filtered working set, clusters the filtered sets into draft gene families and creates a JGI Genome Portal with tools for public access and community-driven curation of the annotation^14^,^52^. For *G. stellatum*, which was not sequenced and assembled at JGI, an additional *ab initio* gene prediction that was performed within the AFTOL-project with AUGUSTUS^53^ 2.3.1 was included in the JGI annotation pipeline.

### Phylogenomic placement of *Cenococcum geophilum*

For phylogenomic placement, we selected additional 48 species (Supplementary Data 1) of which the genomes were available on the basis of the following criteria: all published Dothideomycetes, one to five members of other ascomycetous orders and eight Basidiomycota from five orders selected to cover major orders as well as based on the diversity of lifestyles (biotrophic/hemibiotrophic/necrotrophic pathogens, white rot/brown rot/litter/aquatic saprotrophs and ericoid/ECM species). Genome-scale phylogenetic analyses were conducted with the HAL pipeline^54^. Briefly, orthologous clusters (OC) were identified using MCL across inflation parameters 1, 2, 3 and 5. OCs were filtered to retain non-redundant, single-copy clusters (one protein per genome) with a minimum of 75% representation of the sampled genomes. The resulting 559 single-copy protein clusters were aligned individually in MUSCLE with default settings, after which poorly aligned regions were identified using Gblocks (using liberal setting) and excluded. Aligned proteins were concatenated into a superalignment that included 119,236 amino acid positions and analysed with RAxML v 7.2.6 using the PROTGAMMAWAG model of evolution with 100 bootstrap replicates.

### Estimated ages of the most recent comment ancestors

A chronogram for the 51 taxa RAxML phylogeny was constructed using R8S V1.8 (ref. 55). Estimated mean ages for the most recent comment ancestors of Ascomycota (518 MYA) and Basidiomycota (521 MYA) from Floudas *et al*.^56^ were used as calibration points. Calibration points were cross-validated as follows: method=PL (penalized likelihood) crossv=yes fossilconstrained=yes cvstart=0 cvinc=0.50 cvnum=6 algorithm=TN (truncated Newton). These calibration points passed cross validation and identified a best smoothing parameter value of 10. Reported dates (Supplementary Fig. 3) were inferred using the following parameters: divtime method=PL algorithm=TN; set smoothing=10 penalty=log minRateFactor=0.5 checkgradient=yes.

### Comparative analyses and annotation of functional categories

Multigene families were predicted from 567,845 gene models of the studied 51 species using the MCL algorithm implemented in the JGI clustering pipeline as detailed in Ohm *et al*.^13^. Multigene families were analysed for evolutionary changes in protein family size using the CAFE program^57^. To allow comparing specific functional categories among the selected 51 genomes, the predicted gene models were functionally annotated using the same pipeline. These gene categories included: repeated elements, CAZymes, secreted proteins and genes from the secondary metabolism. RepeatScout^58^ was used to identify *de novo* repetitive DNA in genome drafts. The default parameters (with l=15) were used. RepeatScout generated libraries of consensus repeated sequences that were then filtered as follows: (1) all the sequences less than 100 bp were discarded; (2) repeats having less than 10 copies in the genome were removed (as they may correspond to protein-coding gene families) and (3) repeats having significant hits to known proteins in Uniprot (http://www.uniprot.org/) other than proteins known as belonging to TE were removed. The consensus sequences remaining were manually annotated by a TBLASTX search against RepBase version 19.05 (http://www.girinst.org/repbase/index.html). Using the final libraries of repeated elements, the number of repeat occurrences and the percentage of genome coverage were assessed by masking the genome assemblies using RepeatMasker (http://www.repeatmasker.org). To identify full-length long terminal repeats (LTR) retrotransposons, a second *de novo* search was performed with LTR_STRUC^59^. The putative full-length LTR retrotransposons were annotated manually by a TBLASTX search against RepBase (http://www.girinst.org/repbase/index.html) to identify those belonging to *copia* LTR and to *gypsy* LTR. MISA (http://pgrc.ipk-gatersleben.de/misa/download/misa.pl) with default parameters was used to identify mono- to hexanucleotide simple sequence repeat (SSR) motifs using default parameters. Tandem repeats finder^60^ was used to identify minisatellites (motif between 7 to 100 bp) and satellites (motif above 100 bp) with the following parameters: 2, 7, 7, 80, 10, 50, 500 (Supplementary Data 2). Annotation of carbohydrate active enzyme (CAZyme) genes was performed using the CAZy database (www.cazy.org) annotation pipeline^61^. To assess differences in the numbers of genes present within CAZy families among phylogenetic groups or lifestyles, we applied non-parametric median tests in SPSS (version 23, IBM) with false discovery rate (FDR) correction. The secretome was identified using a custom pipeline as outlined in Kohler *et al*.^1^. Proteins predicted to contain a signal-peptide, but not predicted to localize within the endoplasmic reticulum or mitochondria, and displaying less than one transmembrane domain were considered as secreted proteins. They were classified into four functional categories: CAZymes, lipases, proteases and small-secreted proteins (SSP; proteins less than 300 amino-acids long with no CAZyme, protease or lipase domains). The secreted proteases were identified in the genome using the MEROPS peptide database (http://merops.sanger.ac.uk) and the secreted lipases using the Lipase engineering database (www.led.uni-stuttgart.de). Genes and gene clusters involved in secondary metabolism were predicted using a custom pipeline based on the SMURF method^20^. Conserved protein domains were predicted using PFAM version 27 (ref. 16). SMURF parameter *d* (maximum intergenic distance in base pairs) was set at 3,000 bp, and SMURF parameter *y* (the maximum number of non-secondary metabolism genes upstream or downstream of the backbone gene) was set at 6.

Gene models of major intrinsic proteins (aquaporins) were identified using the automatic annotation pipeline of JGI. The primary structure of the deduced proteins was manually inspected by motive analysis and all models containing less than five or more than 10 transmembrane predictions (TMHMM; http://www.cbs.dtu.dk/services/TMHMM-2.0) were removed. The deduced protein sequences were initially analysed using ClustalW and then applied to a Bayesian approach on the basis of Markov chain Monte Carlo (MCMC) as implemented in the computer program MrBayes 3.2.2 (ref. 62) to estimate phylogenetic relationships. Rather than specifying an amino acid substitution model, we allowed the Markov processes to sample randomly from the substitution models implemented in MrBayes. Trees were sampled every 200 generations, resulting in an overall sampling of 20,000 trees per run, from which the first 4,000 trees of each run were discarded (burn-in). The remaining 12,000 trees sampled in each run were pooled and used to compute a majority rule consensus tree to obtain estimates of the posterior probabilities. Branch lengths were averaged over the sampled trees.

To identify sex-related genes and genes involved in fruiting body formation, we used a combination of BLASTP and TBLASTN searches with known genes of ascomycetous species for mating-type, pheromones and pheromone receptors as well as genes involved in fruiting body development within the predicted proteomes and genomes of *C. geophilum* and its closest relative *G. stellatum*.

### Transcriptome analyses of *Cenococcum geophilum*

Total RNA was extracted from free-living mycelium grown on agar Petri dishes containing Cg-Medium^48^ and covered by a cellophane for 2 months and from ECM root tips of *C. geophilum/Pinus sylvestris* (growth conditions see below) using the RNAeasy Plant Mini Kit of Qiagen with DNase step and addition of 20 mg ml^−1^ polyethylene glycol to the RLC extraction buffer. RNA sequencing was performed using 2 × 100 bp Illumina HiSeq sequencing after mRNA library construction by IGA Technology services (Udine, Italy). Raw reads were trimmed and aligned to the *C. geophilum* reference transcripts (http://genome.jgi-psf.org) using CLC Genomics Workbench 6 and 7 (http://www.clcsupport.com/clcgenomicsworkbench/900/index.php?manual=RNA_Seq_analysis.html) and as described before^1^. To identify differentially regulated transcripts in mycorrhizal tissues compared with free-living mycelium, the test of Baggerly *et al*.^63^ implemented in CLC Genomic Workbench and the Benjamini and Hochberg multiple-hypothesis testing to correct for FDR was applied to the data. If not otherwise indicated, transcripts with ≥5 fold change and a FDR-corrected *P* value <0.05 were kept for further analysis. To assess whether symbiosis-regulated transcripts were either conserved among fungal species or lineage-specific, their protein sequences were queried against the proteins of 50 fungal genomes (Supplementary Data 1) using BLASTP as indicated in Kohler *et al*.^1^. The distributions of the symbiosis-regulated transcripts within each cluster were quantified according to their putative function as CAZymes, SSPs, with KOG classification or without KOG. The enrichment of secreted proteins and SSPs in symbiosis-regulated transcript clusters was assessed by comparing their percentages in symbiosis-regulated transcript clusters to their relative percentage in the total gene repertoire and applying a Fisher's exact test. The data were visualized and hierarchically clustered by the binary distance metric and ward clustering for the analysis of five genomes (Fig. 4) and the euclidean distance metric and the average-linkage clustering method for the 50 fungal genome comparison (Supplementary Fig. 6) using R (package HeatPlus).

### Functional analyses of aquaporins in *Cenococcum geophilum*

For heterologous expression of selected aquaporins in *Xenopus laevis* oocytes, total RNA was isolated from *C. geophilum/P. sylvestris* ECM root tips using the NucleoSpin RNA Plant kit (-NAGEL GmbH, Düren, Germany) with a DNAse step according to the manufacturer's instructions. First-strand cDNA was synthesized in a total volume of 20 μl, containing 50 pmol oligo-d(T)18-primer (Eurofins-MWG-Operon, Ebersberg, Germany), 0.5 μl RiboLock RNase inhibitor (40 U μl^−1^) and 200 U RevertAid Premium Reverse Transcriptase (Thermo Fisher Scientific, Waltham, MA, USA) according to the manufacturer's instructions. The coding regions of AQP genes were PCR amplified in a total volume of 20 μl from 0.2 μl first-strand cDNA using a proof-reading DNA polymerase (Phusion, New England Biolabs, Beverly, MA, USA) according to the manufacturer's instructions with the gene-specific primers (Eurofins) named by the JGI protein IDs (Cenge3:690706, Cenge3:604158, Cenge3:647346 Supplementary Data 15). The coding regions of the selected AQPs were PCR amplified using a proof-reading DNA polymerase (Phusion, New England Biolabs) using the gene-specific forward primer and the pJET1.2 reverse primer (5′-GCGGATAACAATTTCACACAGG-3′). PCR fragments were digested with *XbaI* (New England Biolabs), purified using the NucleoSpin PCR Clean-up kit (MACHEREY-NAGEL GmbH) and cloned into the *SmaI* and *XbaI* (Thermo Scientific) double-digested oocyte expression pGemHE^64^. A minimum of two independent cDNA clones were sequenced. DNA fragments containing the T7-promotor upstream of the open reading frames of AQP coding regions were PCR amplified from pGemHE constructs using M13-primers and OneTaq polymerase (New England Biolabs) and purified by ethanol precipitation. cRNA was synthesized in a total volume of 20 μl using the mMESSAGE mMACHINE T7 Kit (Thermo Fisher Scientific) according to the manufacturer's instructions. *X. laevis* oocytes were collagenase A (5 mg ml^−1^, Roche, Basel, Switzerland) treated in CaCl_2_-free buffer OR-2 (82.5 mM NaCl, 2 mM KCl, 1 mM MgCl_2_, 5 mM HEPES, pH 7.4) under agitation for 30 min at 37 °C to remove the follicle cell layer. Maturity stages V and VI were selected and oocytes were stored for 16 h in iso-osmotic ND96 buffer (96 mM NaCl, 2 mM KCl, 1.8 mM CaCl_2_, 1 mM MgCl_2_, 5 mM HEPES, pH 7.4) before injection. Either 50 nl ND96 buffer (negative control) or 50 nl (5 ng) AQP cRNA (*C. geophilum*: Cenge3:647346, Cenge3:604158, Cenge3:690706 or *Laccaria bicolor*: Lacbi1:392091) were injected using a ‘NanoJect II'-microinjector (Drummond scientific company, Reutling, Germany) and oocytes were stored for further 2 days in ND96 with daily buffer exchange. Oocytes were then transferred into hypotonic buffer (ND96 buffer diluted with water 1:3, 125 mOsm) and swelling was monitored with a video camera (DFK 41BU02, The Imaging Source, Bremen, Germany) for a period of 75 s (one image each 5 s) under a dissection microscope (50-fold magnification). Changes in surface size of single oocytes was determined from single images by ImageJ 1.47v (Wayne Rasband, National Institutes of Health, USA) and used for calculation of *P*_f_ values^64^. At least 20 replicates were made for every construct and the respective controls. (Protocol of animal housing and proceedings of surgically removal of pieces of *X. laevis* female ovary approved by ‘Der Senator für Arbeit, Frauen, Gesundheit, Bahnhofsplatz 29, D-28195 Bremen').

### Gene expression of aquaporins in fungal tissues

To compare the gene expression of the AQPs in free-living mycelium (FLM) and ECM root tips of *C. geophilum/P. sylvestris* ECM plants, following growth conditions were used: FLM was produced in liquid culture containing standard Cg-Medium^48^. Mycelia were grown for 1.5 months in Erlenmeyer flasks and three independent replicates (flasks) were harvested, shock-frozen in liquid N, pulverized and stored at −80 °C until RNA extraction. ECM plants were produced as follows: Mycelium was grown in liquid culture with Cg-Medium^48^ for 2 months before it was fragmented and homogenized using a blender for inoculation. *Pinus sylvestris* seedlings were sawn in pots (300 ml) containing a 1:2 double-autoclaved mixture of quartz sand and sieved forest topsoil. The pots were placed in a growth chamber with day/night cycles of 15 h/9 h, temperature of 26 °C/20 °C, respectively, relative humidity of 60%, and mean irradiance of 750 lux during the day cycles. They were watered every third day. After 2 months, the seedlings were excavated and the fragmented fungal inoculum was sprinkled over the roots of two individuals per pot by means of a pipette. The seedlings were re-potted into the same substrate and pot and cultivated for another 3 months with the same conditions before harvesting. To collect ECM, the roots were washed under tap water, placed in cooled water and ECM root tips were sampled under a binocular loupe using forks and immediately put in tubes that were placed in liquid nitrogen. Tubes were stored at −80 °C until RNA extraction. For each trial, at least three independent replicates corresponding to pots were collected and extracted.

### Drought stress experiment with *mycorrhizal pine* seedlings

A drought stress experiment was set up to assess (1) the impact of drought on AQP expression of *C. geophilum* in ECM of *P. sylvestris* and (2) the effect of mycorrhization on the plant condition under drought. Mycorrhizal and non-mycorrhizal plants were produced as indicated above except for using a commercial container soil (www.oekohum.ch) instead of the forest topsoil showing a pH of 5.8 and plant available nitrogen and phosphorus content of 260 and 180 mg l^−1^ soil, respectively. Six months after fungal inoculation of the seedlings, half of the plants were subjected to a drought-stress and rewatering treatment. Although the control treatments continued to receive water every third day to soil water saturation (approx. 75 ml of water), the drought-stressed seedlings were not watered for 2 weeks and then rewatered for 3 days by applying 75 ml of water the first day and half of it every next day. Soil volumetric water content was followed using 5TE Soil moisture, temperature and electrical conductivity sensors (Decagon Devices) in four pots. It dropped from approximately 25% (corresponding to soil water saturation) to 5% at the first harvesting time point after 10 days to 1% at the second harvesting point after 14 days and retrieved 25% two days after rewatering. AQP expression in ECM tips was assessed at three time points: 10 and 14 days of drought stress and 3 days after rewatering. For each time point, 16 pots were harvested, 4 of each combination (non-mycorrhizal/mycorrhizal, drought-stressed/control). Following plant parameters were assessed during the time course of the experiment: Net photosynthesis, stomatal conductance and transpiration using a LI-6400XT Portable Photosynthesis System (LI-COR) at five time points (8, 9 and 13 days of drought, 1 and 2 days after rewatering). These non-destructive measurements were performed the day before the harvesting time points as well as at two additional days to follow the time course. Plant water use efficiency was calculated as net photosynthesis/transpiration. The pre-dawn water potential of shoots was measured using a M 600 Scholander Pressure Chamber (MMM tech support) at pre-dawn of the three harvesting days. Shoot and root dry weight was assessed and C/N analysis of shoots was performed using the NC 2,500 elemental analyzer of CE instruments. At harvesting days, each pot was checked for the mycorrhization with *C. geophilum* and potential contaminants of other ECM fungi. No contaminating ECM fungi were present in NM and ECM pots. A subsample of roots was assessed for the degree of colonization by *C. geophilum* by counting ECM and NM root-tips. It ranged between 10 and 50% with a mean colonization of 23% in inoculated pots and 0% in NM-pots. ECM root-tips were harvested as described above for RNA-extraction using the NucleoSpin RNA Plant kit (MACHEREY-NAGEL) or the Power Plant RNA isolation kit (MOBIO) as indicated by the providers but with adding 20 mg of polyethylene glycol to the extraction buffer.

### Drought stress experiment of mycelium on agar Petri dishes

AQP expression in drought-stressed FLM was measured on inoculated agar Petri dishes, which were produced as outlined above. The cultures were grown for 12 weeks in a climate chamber (25 °C) after which the cellophanes carrying the mycelia were transferred on new agar dishes. They were grown for another 10 days before starting the drought stress for half of the plates by opening the Petri dishes in a running sterile bench for 24 h, whereas control plates were kept closed and sealed with parafilm. Four plates of each treatment were then harvested by peeling the mycelia from the membranes and immediately shock freezing them in liquid N. The rest of the dishes that were opened for 24 h were closed again but without sealing with parafilm and kept for another 3 days in the running sterile bench. After this time period, the agar was completely dried out (Supplementary Fig. 9) and four plates each of control and drought-stressed mycelia were harvested and kept at −80 °C before RNA extraction. RNA was extracted using the NucleoSpin RNA Plant kit (MACHEREY-NAGEL) or the Power Plant RNA isolation kit (MOBIO) as indicated by the providers but with adding 20 mg of polyethylene glycol to the extraction buffer.

### Gene expression analyses using qPCR

To quantify AQP expression, we applied the absolute quantification method with standard curves using an ABI 7500 Fast real-time cycler as indicated by the provider (Applied Biosystems). RT was performed with 20–250 ng of total RNA (mean of 140 ng and 30 ng for mycelia and mycorrhizas, respectively) using the QuantiTect Reverse Transcription Kit (Qiagen) under conditions recommended by the manufacturer. The primers were designed in the 3′ untranslated region if possible and span an exon–exon junction if possible (sequences of the primers and fragments in Supplementary Data 15). Specificity was tested by virtual PCR (FastPCR; primerdigital.com/fastpcr.html) against the genome and by sequencing the amplified fragment. PCR was performed with the power SYBR green PCR master mix (Applied Biosystems) in a reaction volume of 15 μl containing 4–7 ng of cDNA. Cycling was carried out with the following parameters: 10 min activation of AmpliTaq Gold Polymerase at 95 °C, 40 cycles of 15 s denaturation at 95 °C, 60 s annealing and extension at 60 °C, followed by a dissociation step of 15 s at 95 °C, 60 sec at 60 °C, and 30 s at 95 °C to detect primer dimers and nonspecific amplification products. For each primer pair, we determined the PCR efficiency and the dynamic range of PCR by plotting the threshold cycle (*C*_t_) values generated over a range of dilutions against the log input of cDNA amount from diverse samples. Primer efficiencies were also calculated in each qPCR reaction for standard curves. To obtain accurate results, only primer pairs yielding PCR efficiencies of 90–110% (slope of regression between −3.2 and −3.5) were considered. For several samples, reproducibility was checked by diluting cDNA and performing PCR in triplicate within the same plate and in different qPCR reactions and was confirmed to be high. To quantify the transcripts, cDNAs of at least three independent replicates (for example, mycorrhizas of plants grown in different pots, mycelia from different Petri dishes) were diluted and used in qPCR reactions. Copy numbers of the transcripts were calculated from standard curves that were obtained using synthesized single-stranded sense olignonucleotides (Sigma-Aldrich, Switzerland; Supplementary Data 15) specifying amplicons of the selected genes and using the information from the manufacturer to calculate the copy number of the oligonucleotides present in respective volumes. Serial dilutions of the stocks were carried out in duplicate, and dilutions in the range from 10^1^ to 10^8^ copies, depending on the expression profiles of the AQPs, were used in duplicate PCR to generate standard curves. The standard curve was obtained by plotting the logs of the calculated copy number against *C*_t_. The copy numbers of unknown samples were calculated from the regression line. The transcript copy numbers were normalized to 10^4^ actin (JGI ID Cenge3: 653800) transcripts, which was used as internal control.

### Statistics of aquaporin expression and plant parameters

To assess the effect of treatments on AQP expression, we applied linear ANOVA in SPSS statistics (IBM). If necessary, expression values were log10 transformed to meet the assumption of normality. The effect of the drought/rewatering treatment on AQP expression at single time points was tested by the Kruskal–Wallis *H* statistics in SPSS (IBM). We applied correlation analyses between AQP expression and diverse plant parameters in SPSS (IBM) using Pearson's coefficient and two-tailed significance testing.

To assess the effect of mycorrhization and drought on the shoot water potential, needle nitrogen content as well as shoot and root dry weights in the drought/rewatering experiment, we applied two-way or three-way analyses of variance, respectively, including the fixed factors ‘mycorrhization' and ‘drought' and interactions in all the analyses and additionally the factor ‘day' and its interactions for the dependent variable ‘shoot water potential'. For the plant gas exchange parameters, which were measured at 5 days, we applied a linear mixed-effects model applying the restricted maximum likelihood estimation method to take into account the repeated measurements at some days as random factor with pots as subjects and including ‘mycorrhization', ‘drought', ‘day' and their interactions as fixed factors in SPSS (IBM). For the dependent variable water use efficiency, only control plants were taken into account because of the missing values in the drought treatment since not calculable at high drought stress (transpiration=0). Outliers (that is, two Scholander shoot water potential measurements) were removed before the analyses.

### Mycorrhiza-induced plant genes

Double-mycorrhizal *P. sylvestris* seedlings were produced as described in Kipfer *et al*.^65^ using *C. geophilum* 1.58 and the abundant pine symbiotic ECM species *Rhizopogon roseolus* (Corda) Th. Fr. or *Suillus granulatus* (L.) Roussel. Four months after inoculation, three and two replicate plants were harvested for each fungal combination and for non-mycorrhizal plants, respectively. Single fine root tips were collected for each ECM species per plant and for non-mycorrhizal plants in separate tubes and were shock-frozen. Total RNAs were extracted as described above and cDNA was amplified using the SMART PCR cDNA Synthesis Kit (Clontech) according to the manufacturer's instructions.

### Oligoarray construction and hybridization

A 12-plex oligoarray was manufactured by NimbleGen Systems using 43,926 454-sequencing-derived contigs and selected singletons of expressed sequence tags corresponding to ∼7,000 and 5,500 unigenes from *Pinus sylvestris* and *Cenococcum geophilum.* The expressed sequence tags used for microarray construction were produced using the SMART PCR cDNA Synthesis Kit (Clontech) as follows: Two cDNA populations were amplified from *C. geophilum*/*P. sylvestris* ECM sampled from natural forest stand with and without irrigation. Three cDNA populations originated from pure culture mycelia of *C. geophilum* strain 1.22, isolated from sclerotia sampled in June 1980, which were grown on agar petri-dishes containing Cg-Medium^48^ and were subjected to different conditions imposing drought. Total RNAs were extracted from several samples per cDNA population using the RNeasy Plant Mini Kit (Qiagen). For mycelial samples, mRNA was extracted using the Oligotex mRNA kit (Qiagen). Total and mRNA samples were amplified using the SMART PCR cDNA Synthesis Kit (Clontech) according to the manufacturer's instructions and were 454-sequenced. The reads were assembled and cleaned from contaminations (bacteria, rRNA) resulting in 34,304 and 9,622 contigs and singletons, respectively, which were used for the array design. They were assigned to fungal or plant sequences on the basis of BLASTN and BLASTX scores obtained from searches against NCBI databases. The arrays contained three independant, nonidentical 60-mer probes per sequence. For 700 randomly selected probes, technical replicates were present on the array. Single-dye labelling of samples, the hybridization procedures and data acquisition were all performed at the NimbleGen facilities in Reykjavik, Iceland, following their standard protocol. Three and two biological replicates were performed for each condition of ECM and NM, respectively. Microarray probe intensities with fungal or plant assignment were separately quantile normalized across all chips. Average expression levels were calculated for each gene from the independent probes on the array and were used for further analysis. A transcript was deemed expressed when its signal intensity was three-fold higher than the mean signal-to-noise threshold (cut-off value) of randomly designed oligonucleotide probes present on the array (50 to 100 arbitrary units). The maximum signal intensity values were ∼65,000 arbitrary units. Normalized data were subjected to the Cyber-T statistical framework^28^. We used a Bayesian confidence estimate value of 6 and a sliding window of 101 genes for the standard deviation estimation. Transcripts with a significant *P* value <0.05 and a PPDE>0.97 were considered as significantly differentially expressed. Differentially expressed transcripts were annotated in more detail by blasting them against diverse databases such as Swissprot, KEGG, KOG and PFAM as well as by using the MERCATOR tool^66^ for the pine transcripts differentially regulated between ECM fungi. We first blasted these transcripts against the *Picea abies* genome (http://congenie.org/start) and used the unigenes of the best matches for functional annotation by MERCATOR. Functional classification was manually adapted in few cases where several categories were provided by MERCATOR or where gene descriptions indicated a particular function.

We further used GeneANOVA^67^ and Genesis^68^ to perform principal component analysis and produce Heatmaps. For Heatmaps, the expression values were log2 transformed and mean centred by genes.

### Data availability

Genome assemblies and annotations for the organisms used in this study are available via the JGI fungal genome portal MycoCosm^14^ (http://jgi.doe.gov/fungi). In addition, the newly sequenced genome assemblies and annotations have been deposited to GenBank under the following accessions codes/BioProjects: *Cenococcum geophilum*: LKKR00000000/PRJNA196023; *Glonium stellatum*: LKAO00000000/PRJNA295956; *Lepidopterella palustris*: LKAR00000000/PRJNA196014. Amino acid alignment and phylogenetic tree files have been deposited in the TreeBase repository under accession number S19566. The complete transcriptome data sets are available at the Gene Expression Omnibus at the National Center for Biotechnology Information (http://www.ncbi.nlm.nih.gov/geo/). The microarray derived data sets as series GSE80549, the RNA-Seq derived data as GSE83909. The authors declare that all other data supporting the findings of this study are available within the article and its Supplementary Information files or are available from the corresponding authors upon request.

## Additional information

**How to cite this article:** Peter, M. *et al*. Ectomycorrhizal ecology is imprinted in the genome of the dominant symbiotic fungus *Cenococcum geophilum*. *Nat. Commun.* 7:12662 doi: 10.1038/ncomms12662 (2016).

## Supplementary Material

Supplementary Information Supplementary Figures 1-12, Supplementary Tables 1-3 and Supplementary References.

Supplementary Data 1 Species used in this comparative genome study.

Supplementary Data 2 Repeat element content present in the 60 studied genomes.

Supplementary Data 3 Predicted number of genes in C. *geophilum* and other studied Dothideomycetes broken down by the level of conservation.

Supplementary Data 4 Unique protein families in C. *geophilum*.

Supplementary Data 5 Expanded and contracted protein families in C. *geophilum*.

Supplementary Data 6 Number and kind of secreted enzymes present in C. *geophilum* and other studied fungi.

Supplementary Data 7 Plant cell wall degrading enzymes found in C. *geophilum* and other studied fungi.

Supplementary Data 8 CAZyme content in the genomes of C. *geophilum* and other fungi.

Supplementary Data 9 Proteins and protein clusters in the C. *geophilum* genome involved in secondary metabolism.

Supplementary Data 10 Significantly symbiosis-regulated genes in C. *geophilum*.

Supplementary Data 11 Aquaporin content in the genomes of C. *geophilum* and other fungal species.

Supplementary Data 12 Regulated plant genes in mycorrhizal versus non-mycorrhizal P. *sylvestris* root tips.

Supplementary Data 13 Regulated P. *sylvestris* genes in mycorrhizas interacting with C. *geophilum* compared to other mycorrhizal fungi.

Supplementary Data 14 Putative homologs of genes implicated in mating and fruiting body production in C. *geophilum* and G. *stellatum*.

Supplementary Data 15 Sequences of primers and of synthesized DNA fragments used for functional studies of aquaporins.

## Figures and Tables

**Table 1:** The most highly upregulated genes in ectomycorrhizal roots of *Cenococcum geophilum* and *Pinus sylvestris* compared with free-living mycelium.

**Protein ID**	FLM—means*	ECM—means*	**Fold change**	**Size (AA)**	**Definition**	**Predicted function**	**Conservation**†
690706	0.6	3,849.1	**6,133**	283	Aquaporin (major intrinsic protein)	Transport mechanisms	Conserved
600722	0.4	550.1	**1,453**	468	Sugar transporter	Transport mechanisms	Conserved
647346	5.3	5,666.3	**1,073**	316	Aquaporin (major intrinsic protein)	Transport mechanisms	Conserved
697512	0.1	112.5	**1,018**	287	Unknown	No KOG‡	Cenge/Glost/Leppa/Hyspu
654895	1.0	476.0	**484**	209	Peptidase	Metabolism	Conserved
655146	3.7	1,560.8	**420**	195	Unknown	No KOG	Conserved
730139	0.5	185.9	**410**	523	MFS transporter	Transport mechanisms	Conserved
612206	0.3	91.6	**344**	496	MFS transporter	Transport mechanisms	Conserved
333290	0.2	51.8	**341**	165	Unknown	No KOG	Unique
600293	0.3	97.3	**313**	121	Unknown	No KOG	Conserved
698167	2.1	636.3	**297**	259	Unknown	Small secreted protein (SSP)	Unique
616643	0.1	36.0	**279**	93	Unknown	No KOG	Conserved
660401	0.1	15.6	**270**	58	Unknown	SSP	Unique
680403	0.1	21.0	**236**	135	Unknown	SSP	Unique
605087	8.9	2,016.8	**227**	539	MFS transporter	Transport mechanisms	Conserved
610797	0.2	39.1	**225**	567	Amino acid permease	Transport mechanisms	Conserved
608762	0.9	204.9	**222**	269	Glucose/ribitol dehydrogenase	Poorly characterized	Conserved
613185	1.6	363.0	**222**	359	G-protein	No KOG	Overrep Mytil versus Dothis
676136	2.0	409.9	**207**	151	Ricin B lectin	CAZyme (CBM13, plant cell wall)	Cenge/Copci
649427	1.6	325.8	**207**	248	Unknown	No KOG	Conserved

ECM, ectomycorrhizal roots; FLM, free-living mycelium.

^*^Values are means of three replicates; given in RPKM (reads per kilobase of transcript per million reads mapped).

^†^Conservation of proteins: protein orthologues only present in mentioned fungal species; Cenge, *Cenococcum geophilum*; Copci, *Coprinus cinerea*; Glost, *Glonium stellatum*; Hyspu, *Hysterium pulicare*; Leppa, *Lepidopterella palustris*; Unique, protein family unique to *C. geophilum*; Overrep Mytil versus Dothis, over-represented gene family in Mytilinidiales compared with other Dothideomycetes; conserved, protein orthologues present in several orders without particular pattern.

^‡^KOG: EuKaryotic Orthologous Groups (http://genome.jgi-psf.org/help/kogbrowser.jsf).
