# Supplementary material for: Ectomycorrhizal ecology is imprinted in the genome of the dominant symbiotic fungus Cenococcum geophilum
Source: Nat Commun. 2016 Sep 7;7:12662. doi: 10.1038/ncomms12662 (PMC5023957; doi:10.1038/ncomms12662)
Supplement: Supplementary Information — Supplementary Figures 1-12, Supplementary Tables 1-3 and Supplementary References. [file ncomms12662-s1.pdf]

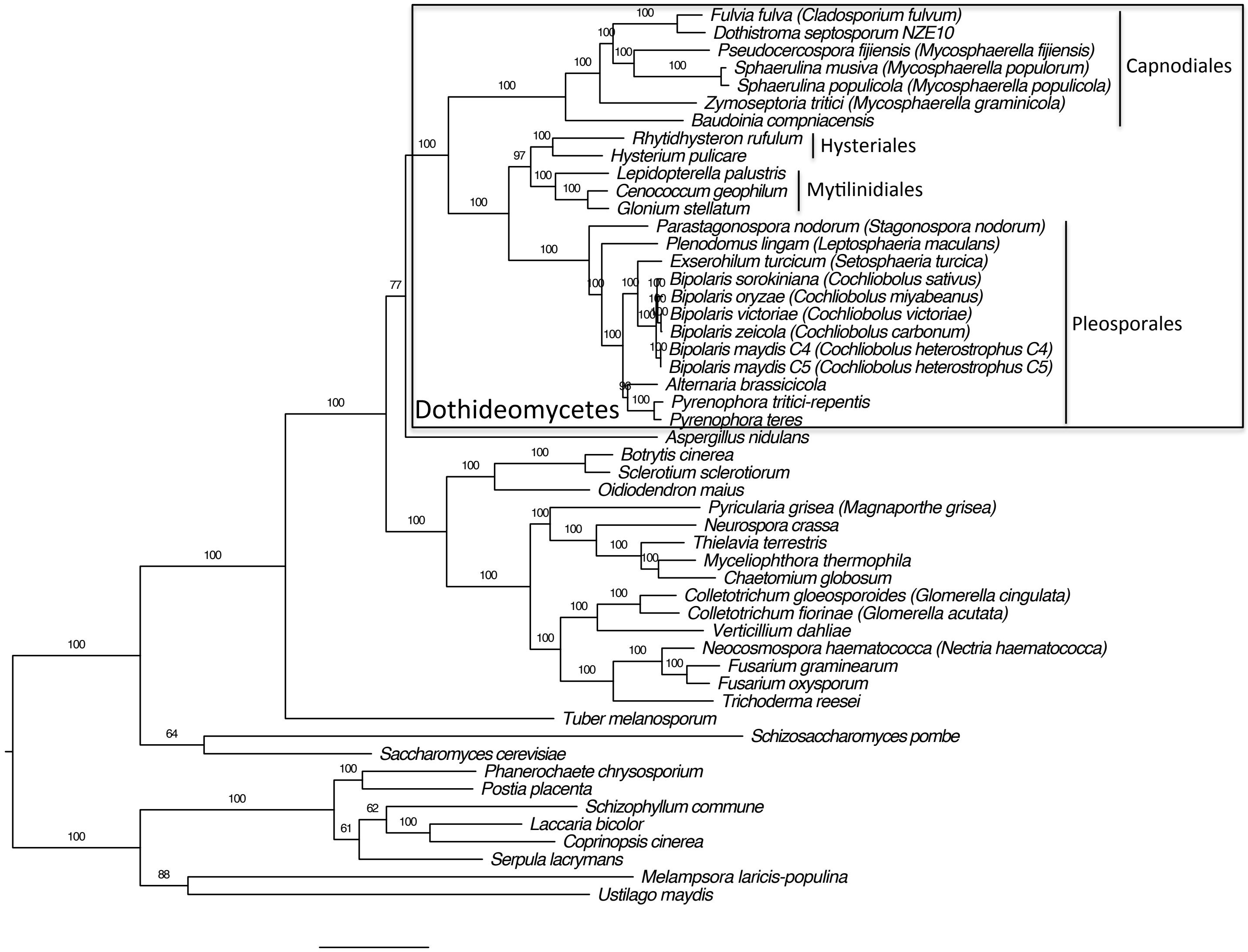

Supplementary Figure 1. Phylogenetic placement of *Cenococcum geophilum, Glonium stellatum* and *Lepidopterella plaustris*. Genomescale phylogenetic analysis based on 559 single-copy gene clusters using RAxML. Bootstrap support for branches are provided. Scale bar of branch length: 0.2.

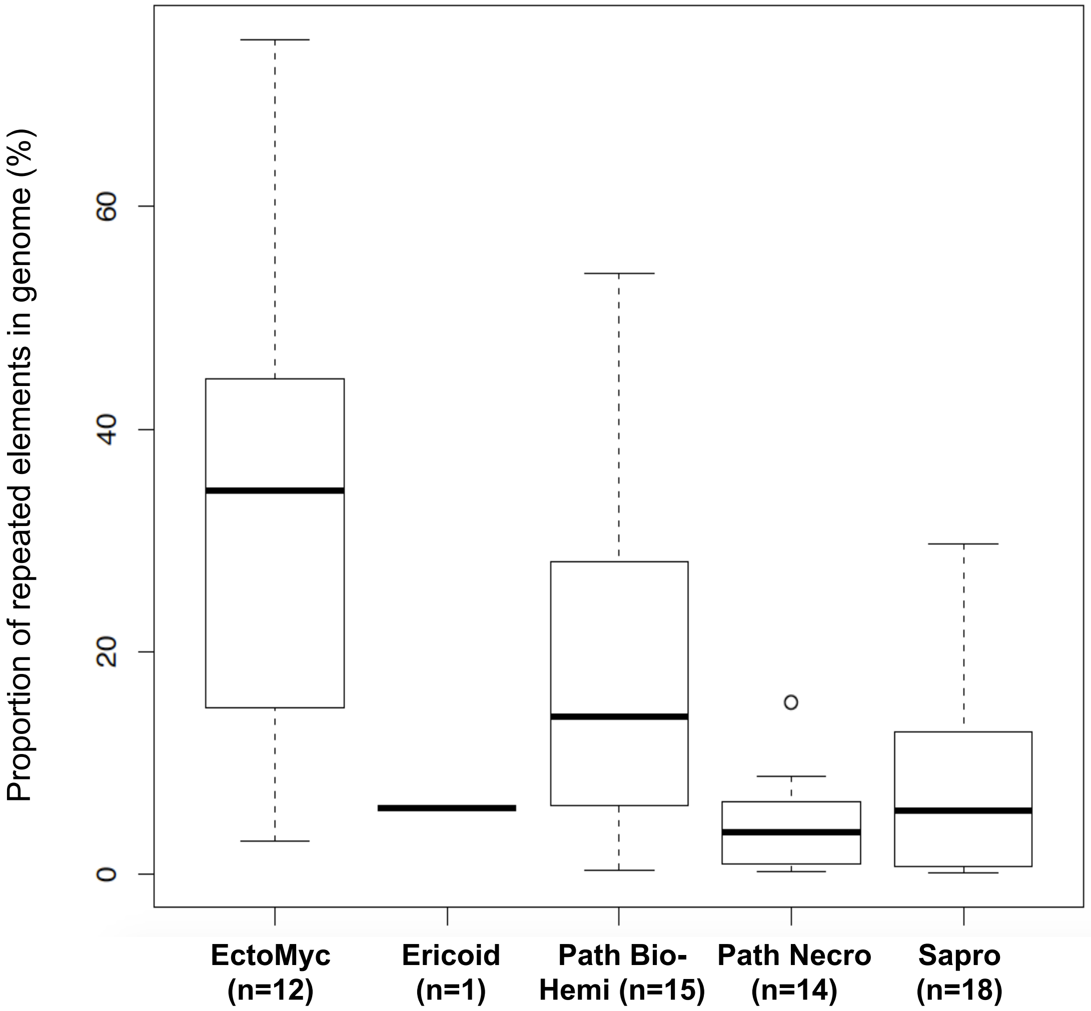

Supplementary Figure 2. Proportion of genome covered by repeated elements in fungi with different lifestyles. Box plots indicate the median, the 25th and 75th percentiles (box), the 5th and 95th percentiles (whiskers) and outliers (dots). In brackets, the number of studied species within each lifestyle is provided. Medians are significantly different according to an independent samples median test performed with SPSS (IBM; Supplementary Data 2). Ectomyc, ectomycorrhizal; Ericoid, ericoid mycorrhizal; Path Bio-Hemi, biotrophic and hemibiotrophic pathogens, Path Necro, necrotrophic pathogens; Sapro, saprotrophs.

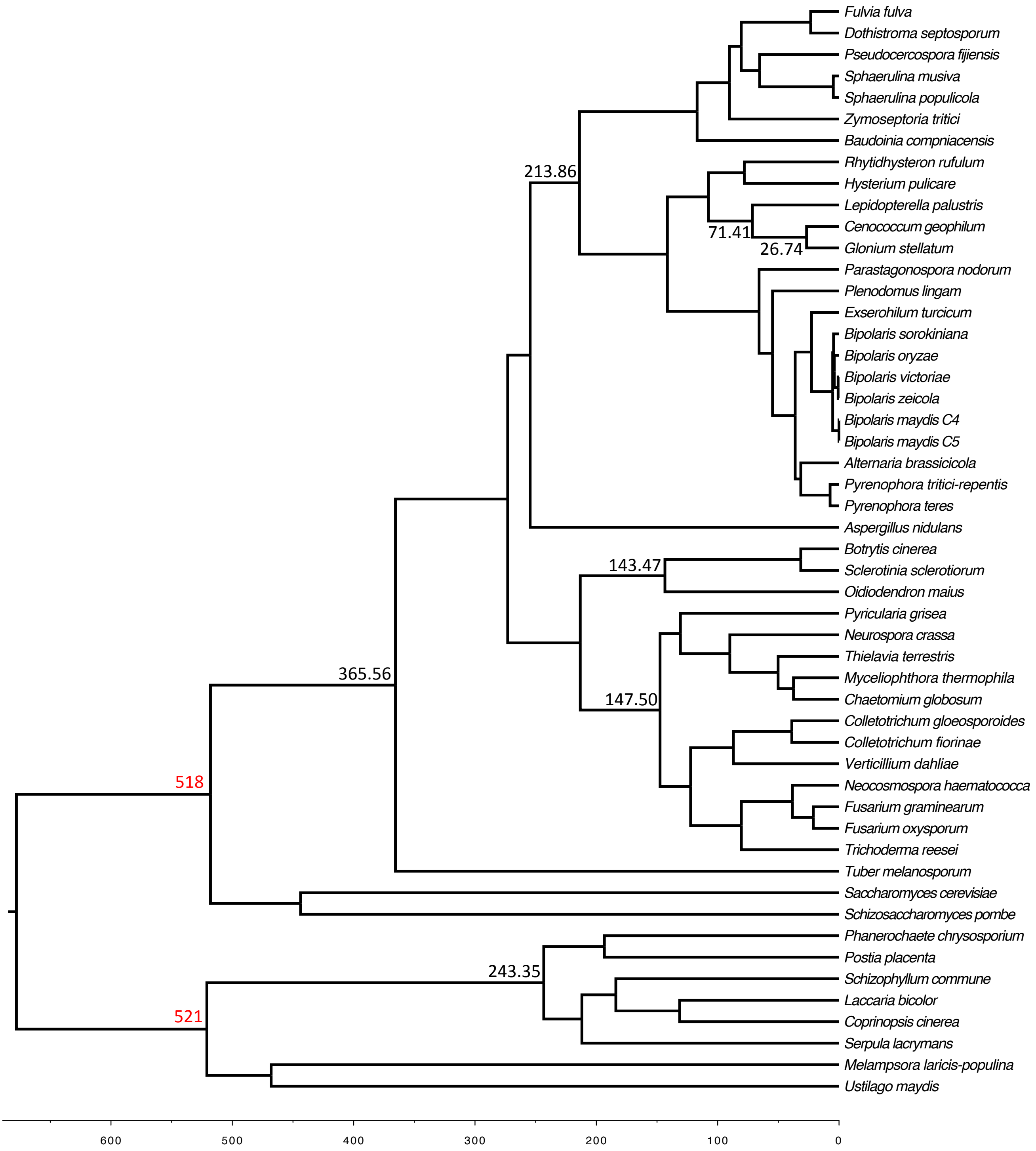

Supplementary Figure 3. Chronogram of RAxML phylongeny. Numbers in red are fixed age calibration points from Floudas *et al.*[1] for Ascomycota and Basidiomycota. Numbers in black are inferred node ages from r8s analyses. All ages are in millions of years.

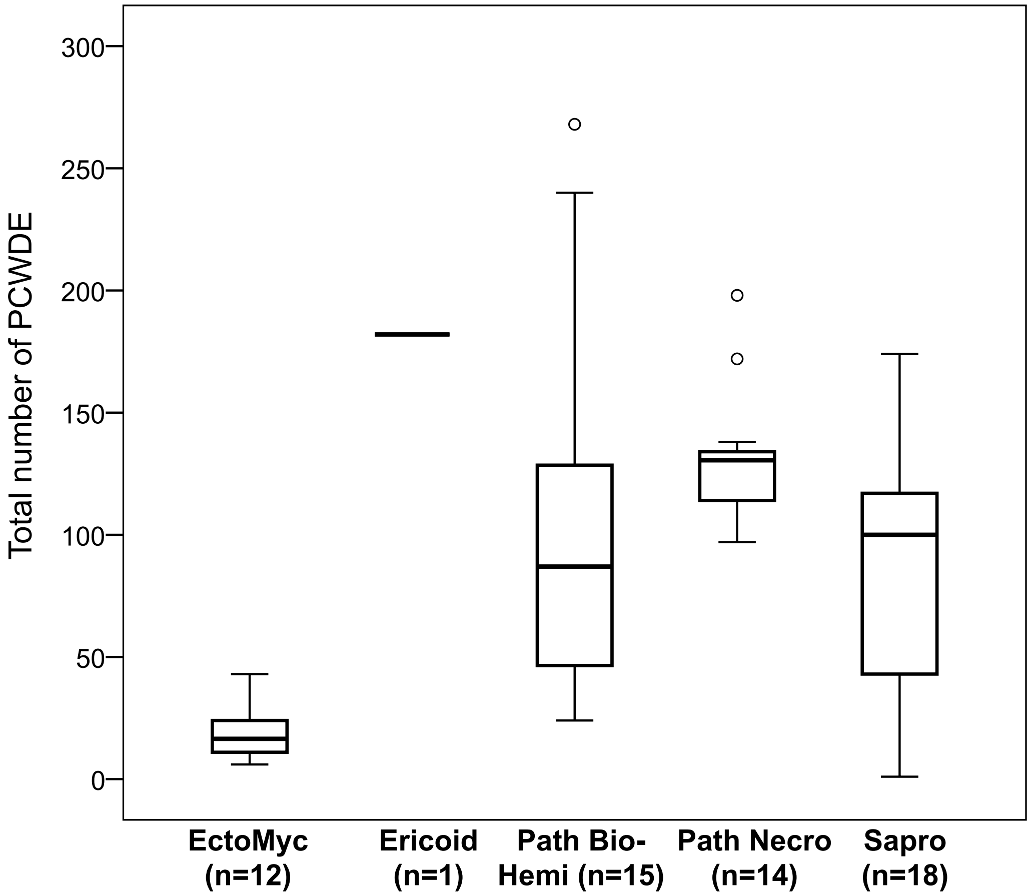

Supplementary Figure 4. Total number of plant cell wall degrading enzymes of fungi with different lifestyles. Box plots indicate the median, the 25th and 75th percentiles (box), the 5th and 95th percentiles (whiskers) and outliers (dots). In brackets, the number of studied species within each lifestyle is provided. Medians are significanly different according to an independent samples median test performed with SPSS (IBM; Supplementary Data 7). PCWDE, plant cell wall degrading enzymes; Ectomyc, ectomycorrhizal; Ericoid, ericoid mycorrhizal; Path Bio-Hemi, biotrophic and hemibiotrophic pathogens, Path Necro, necrotrophic pathogens; Sapro, saprotrophs.

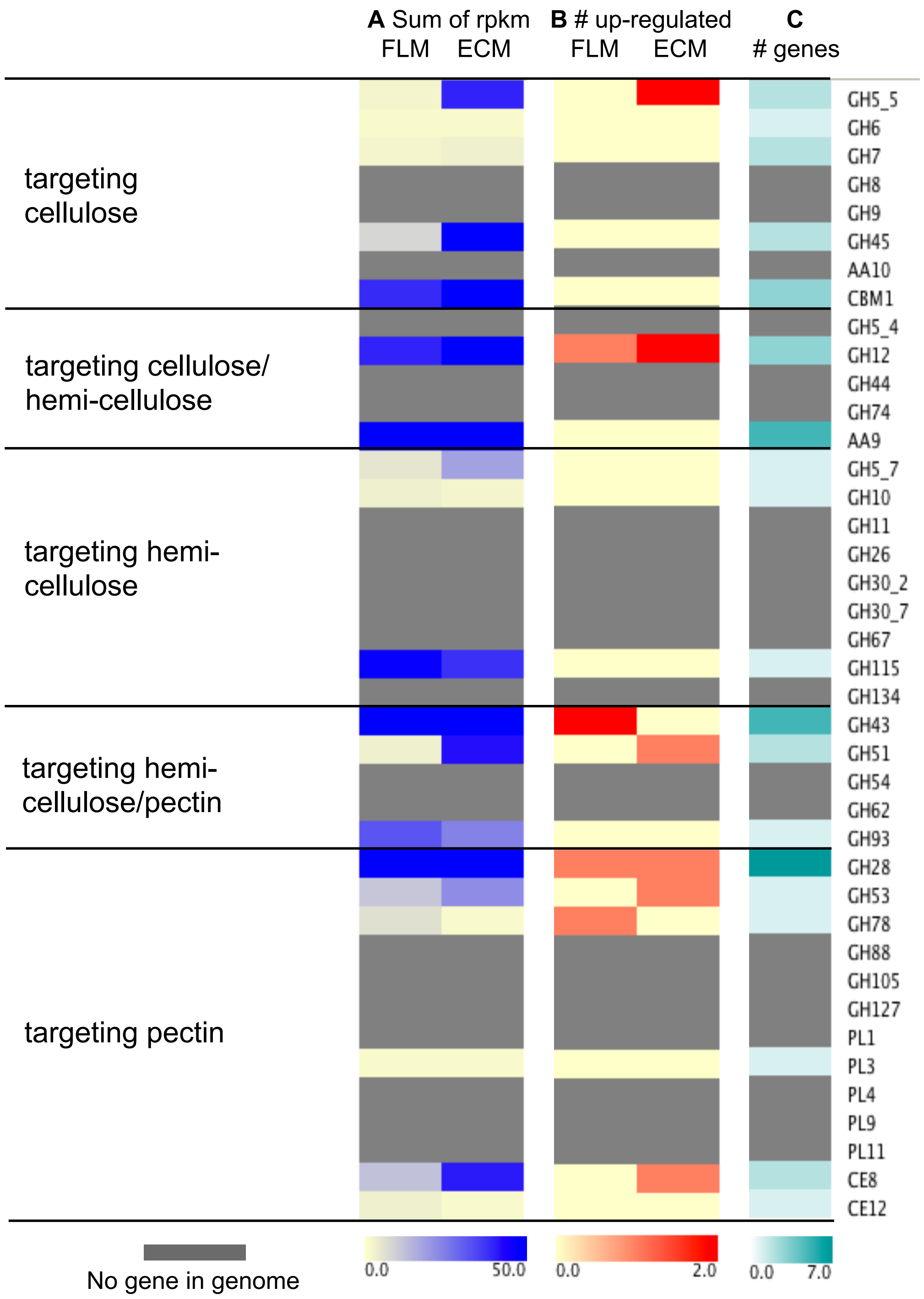

Supplementary Figure 5. Characterization of selected CAZymes of *Cenococcum geophilum*. Expression of CAZymes (A; sum of rpkms) and their up-regulation (B; fold-change>5, FDR-corrected p <0.05) in free-living mycelium (FLM) and mycorrhizal tissue (ECM). The total number of genes identified in the genome for the respective CAZy family is given in C.

a

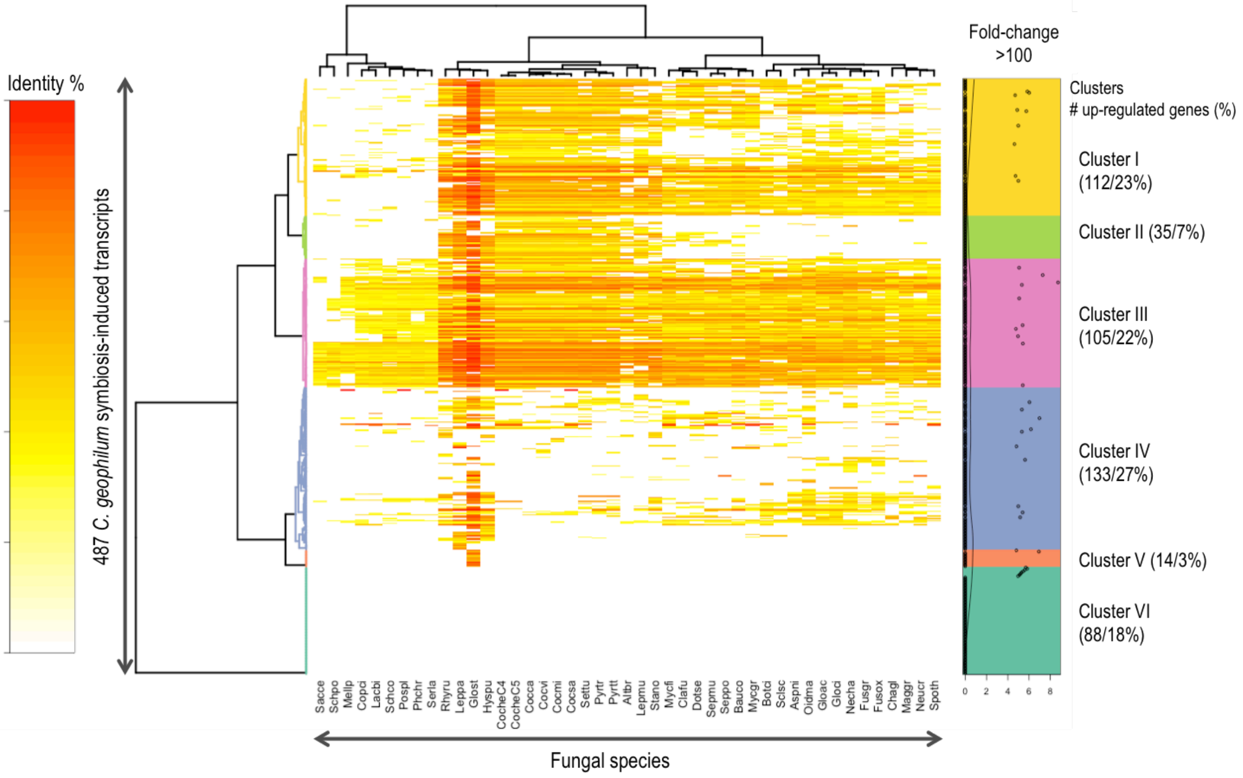

b

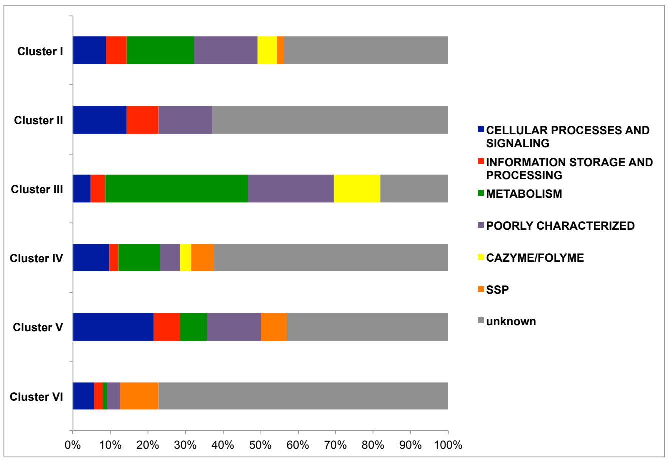

	Secretome (%)	enriched	Fisher **p-value**	SSP (%)	enriched	Fisher **p-value**
**Cluster I**	6	no	0.1666	2	no	0.5167
**Cluster II**	0	no	1	0	no	1
**Cluster III**	7	no	0.1317	0	no	1
**Cluster IV**	7	no	0.08932	6	yes	0.001049
**Cluster V**	7	no	0.4383	7	no	0.1953
**Cluster VI**	11	yes	0.00282	10	yes	8.23E-06
**Total up**	7	yes	0.001358	4	yes	6.60E-05
**Genome**	4			2		

c

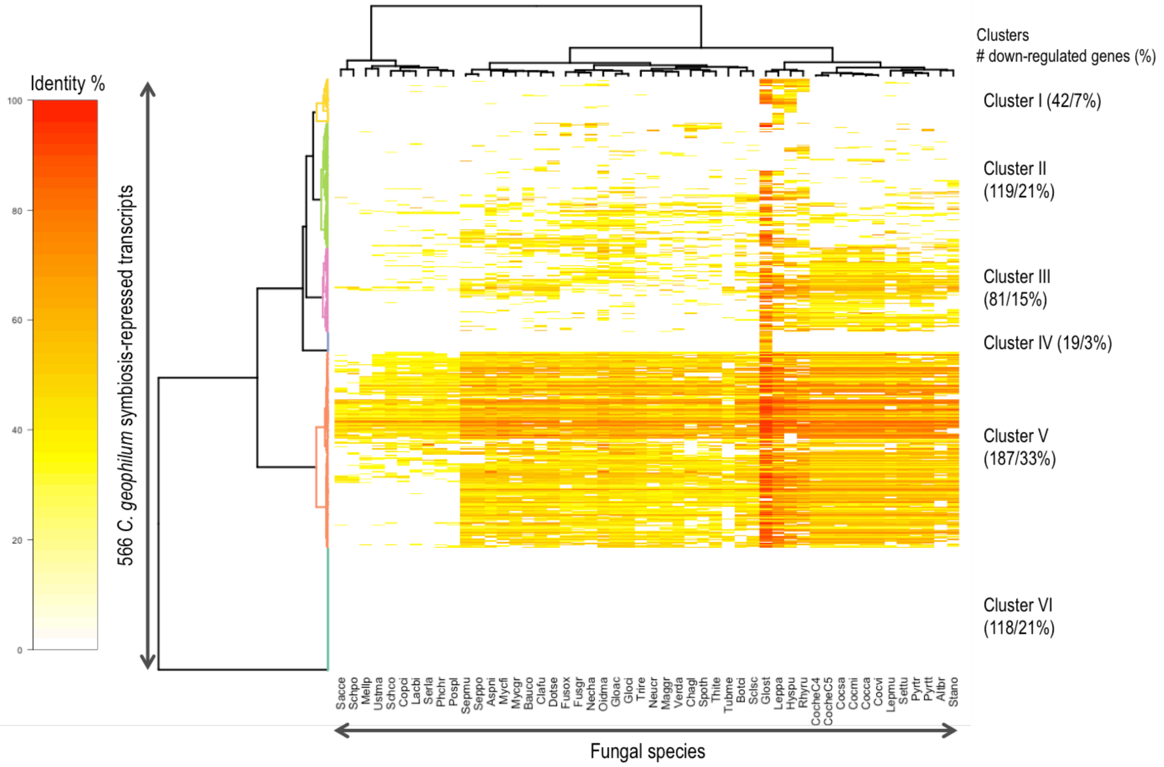

d

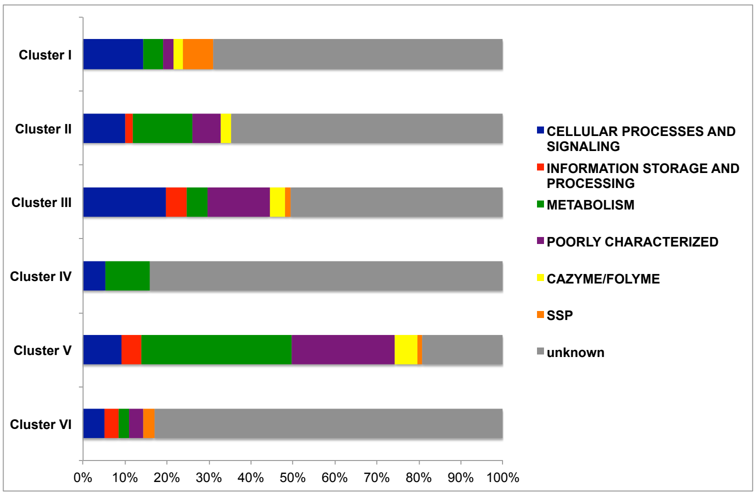

	Secretome (%)	enriched	Fisher p-value	SSP (%)	enriched	Fisher p-value
**Cluster I**	12	no	0.02611	7	no	0.0266
**Cluster II**	6	no	0.2051	0	no	1
**Cluster III**	9	no	0.0448	1	no	0.7163
**Cluster IV**	5	no	0.5429	0	no	1
**Cluster V**	11	yes	6.86E-05	1	no	0.7862
**Cluster VI**	3	no	0.7064	3	no	0.27
**Total down**	8	yes	2.38E-05	2	no	0.51
**Genome**	4			2		

Supplementary Figure 6. Functional analysis of symbiosis-regulated transcripts. The heatmaps depict a double hierarchical clustering of 487 symbiosis-upregulated (**a**) and 566 symbiosis-downregulated (**c**) *Cenococcum geophilum* genes (rows, fold-change >5, FDR-corrected $p<0.05$). Symbiosis-regulated genes were blasted (BLASTP) against 50 fungal genomes to find homologs. Homologs are coloured from yellow to red depending on the percentage of similarity. The heatmaps represent a double-hierarchical clustering of the symbiosis-regulated transcripts orthologs in the 50 fungal genomes. Data were visualized and clustered using R (package HeatPlus). The hierarchical clustering was done by using a binary distance metric and ward clustering method. (**a**) Transcripts upregulated >100-fold are shown as circles on the right panel, next to it is the number of transcripts in each cluster. For each cluster the percentages of putative functional categories are given as bargrams for upregulated (**b**) and downregulated (**d**) genes. A table shows the percentage of transcripts coding for secreted proteins and small secreted proteins in each cluster. A Fisher exact test ($p<0.01$) was applied to test if these categories were enriched compared to the number of these genes in the whole genome (**b and d**).

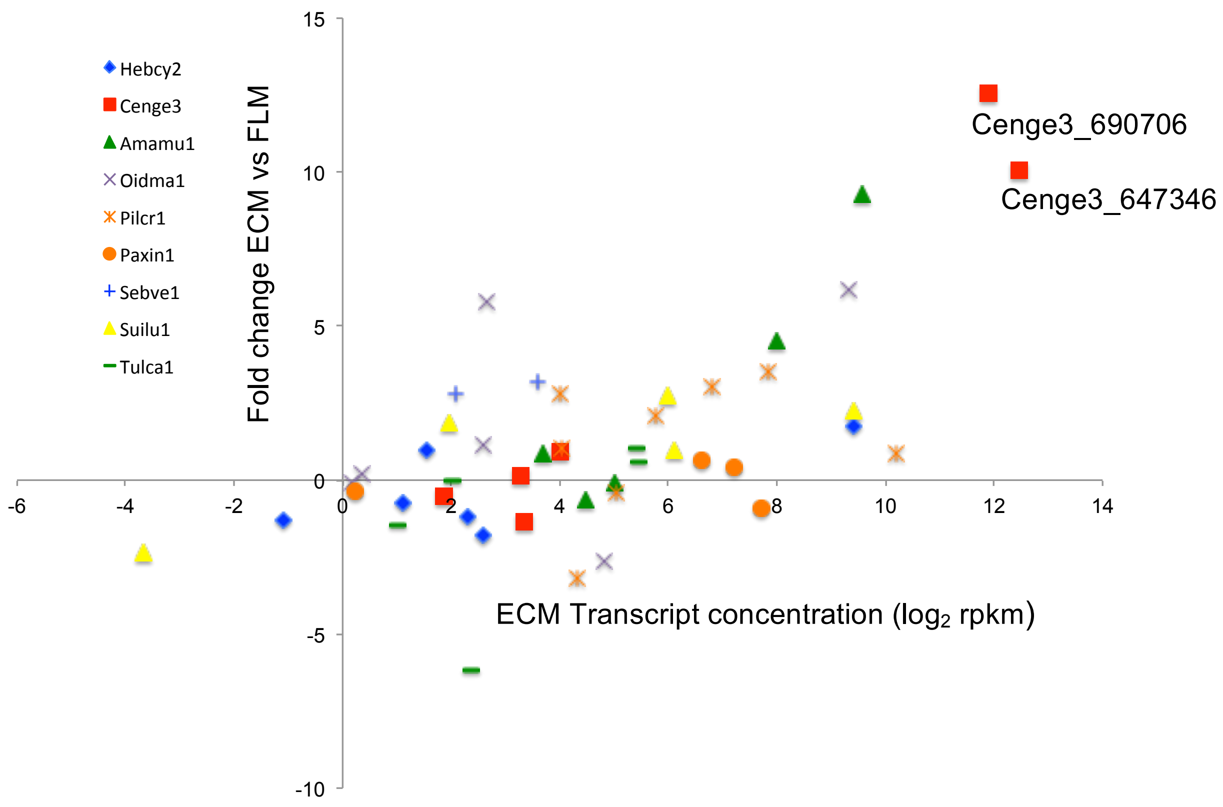

Supplementary Figure 7. Expression and fold-change of aquaporin genes from diverse mycorrhizal fungi in mycorrhizal tissue versus free-living mycelium. For experimental conditions and transcriptome analyses see Kohler *et al.*[2]. ECM; ectomycorrhizal tissue; FLM, free-living mycelium. Ectomycorrhizal species: Hebcy2, *Hebeloma cylindrosporum*; Cenge3, *Cenococcum geophilum*; Amamu1, *Amanita muscaria*; Pilcr1*, Piloderma croceum*; Paxin1, *Paxillus involutus*; Suilu1*, Suillus luteus*; Ericoid mycorrhizal species: Oidma1. *Oidiodendron maius*; Orchid mycorrhizal species: Tulca; *Tulasnella calospora*; Sebve1, *Sebacina vermifera.*

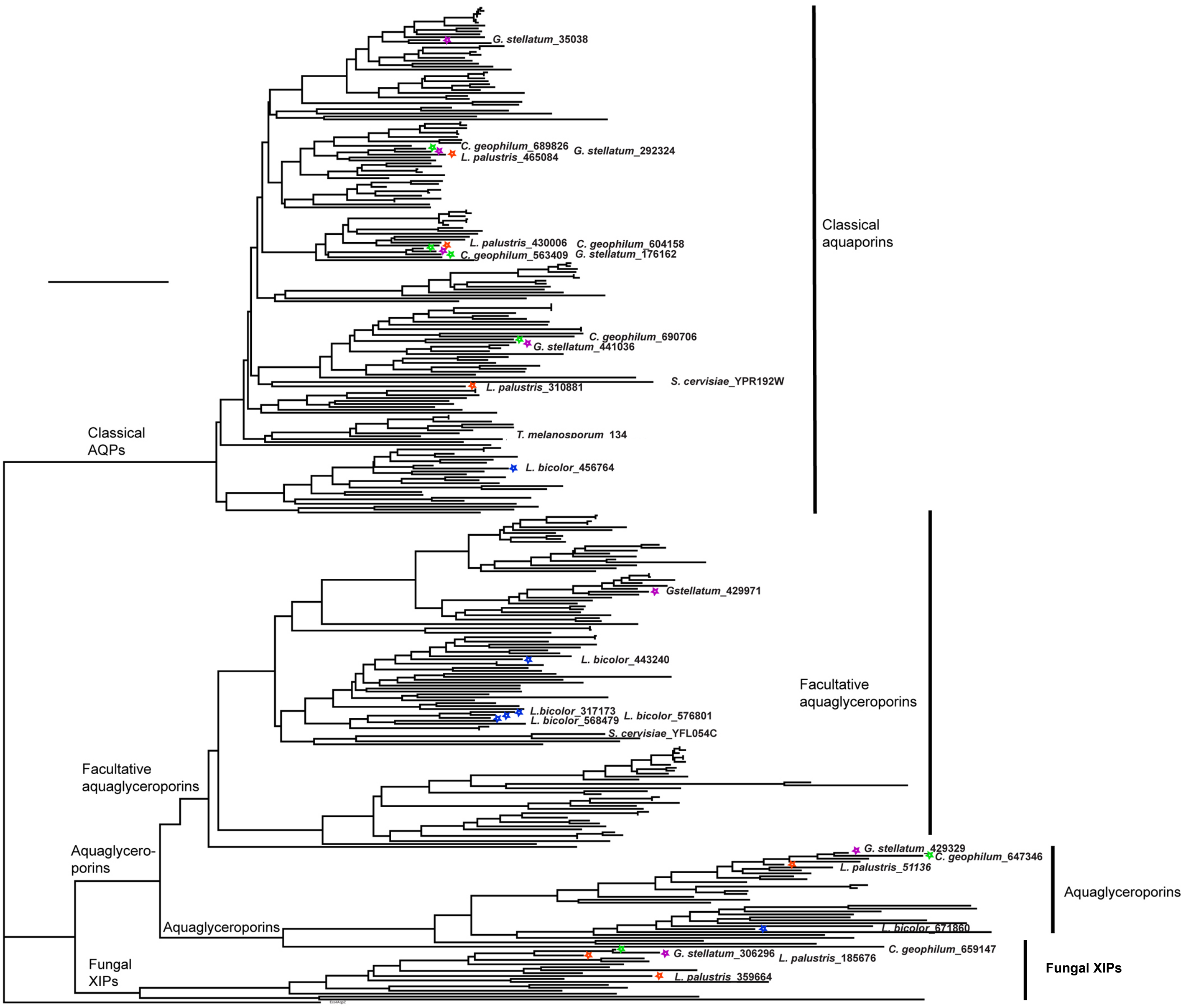

Supplementary Figure 8. Phylogenetic relationship and classification of fungal aquaporins. The deduced protein sequences of *Cenococcum geophilum, Glonium stellatum* and *Lepidopterella palustris* aquaporins (AQPs) were compared with AQP protein sequences of the other studied fungal species (Supplementary Table 1). Scale bar of branch length: 0.2.

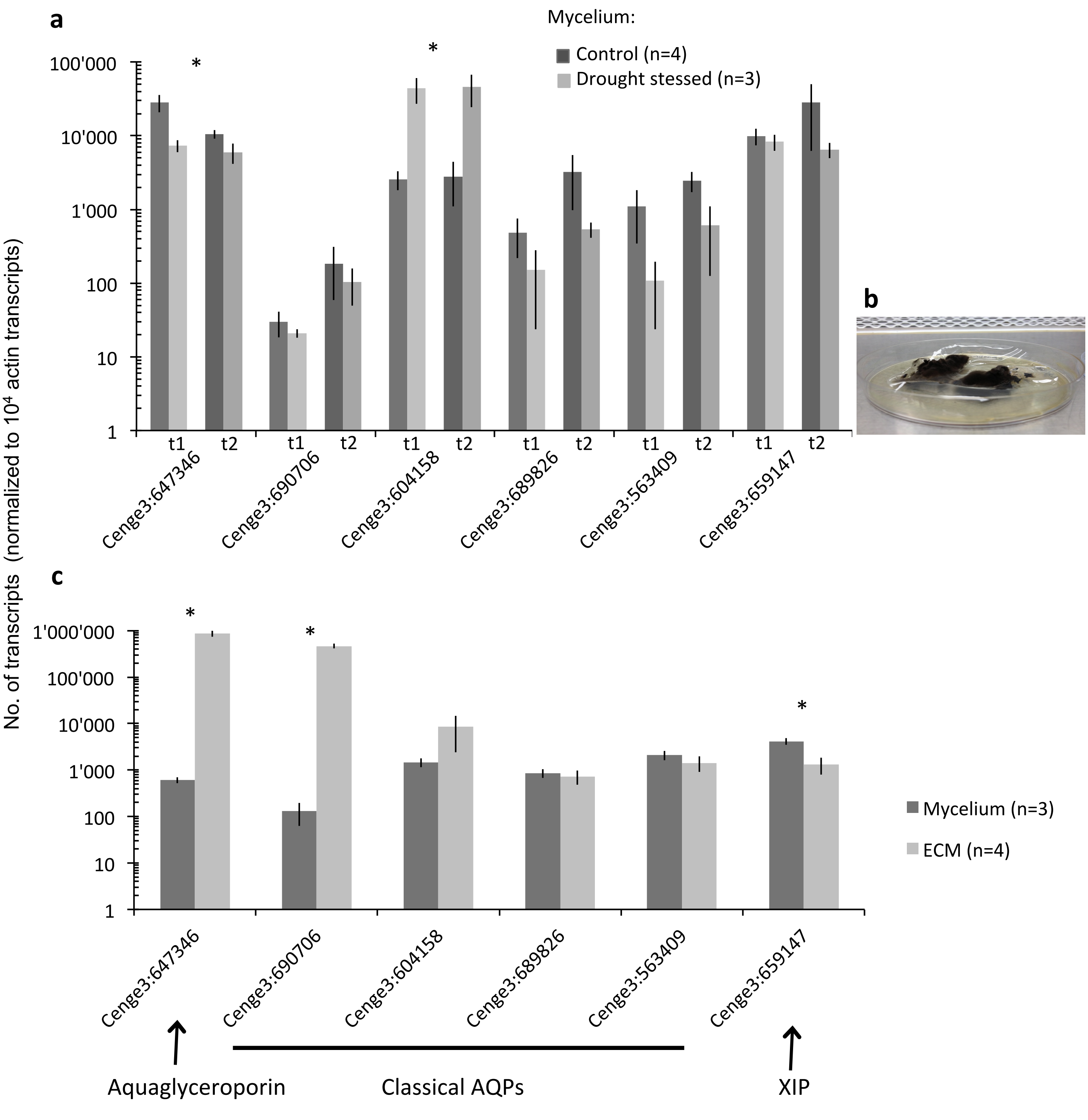

Supplementary Figure 9. Expression of aquaporins in *Cenococcum geophilum*. (**a, b**) Impact of drought on aquaporin (AQP) expression in free living mycelium of *C. geophilum* and (**c**) qPCR validation of symbiosis regulation of the AQPs. (**a**) Expression of aquaporins as assessed by quantitative reverse-transcriptase polymerase chain reaction in free living mycelium (FLM) grown in agar Petri dishes for four months and subjected to drought by opening the Petri dishes for 24h (t1) and growing for another 3 days in re-closed dishes (t2). Significance of the drought treatment as assessed by 2-way analysis of variance with treatment and timepoint as factors are indicated by an asterisk (p<0.05). No significant treatment x timepoint interaction was found. (**b**) *C. geophilum* mycelium on cellophane after 4 days of agar-drying. (**c**) AQP expression in FLM and 3-months-old, functioning ectomycorrhizas (ECM) of *C. geophilum* and *Pinus sylvestris*. Sigificantly symbiosis-regulated AQPs are indicated by an asterisk (p<0.05). Data for each condition are presented as the mean (±s.e.m.) and were obtained from the indicated number of biological replicates (i.e. separate Petri dishes, flasks or separate plants/pots). Details of statistics are provided in Supplementary Table 2.

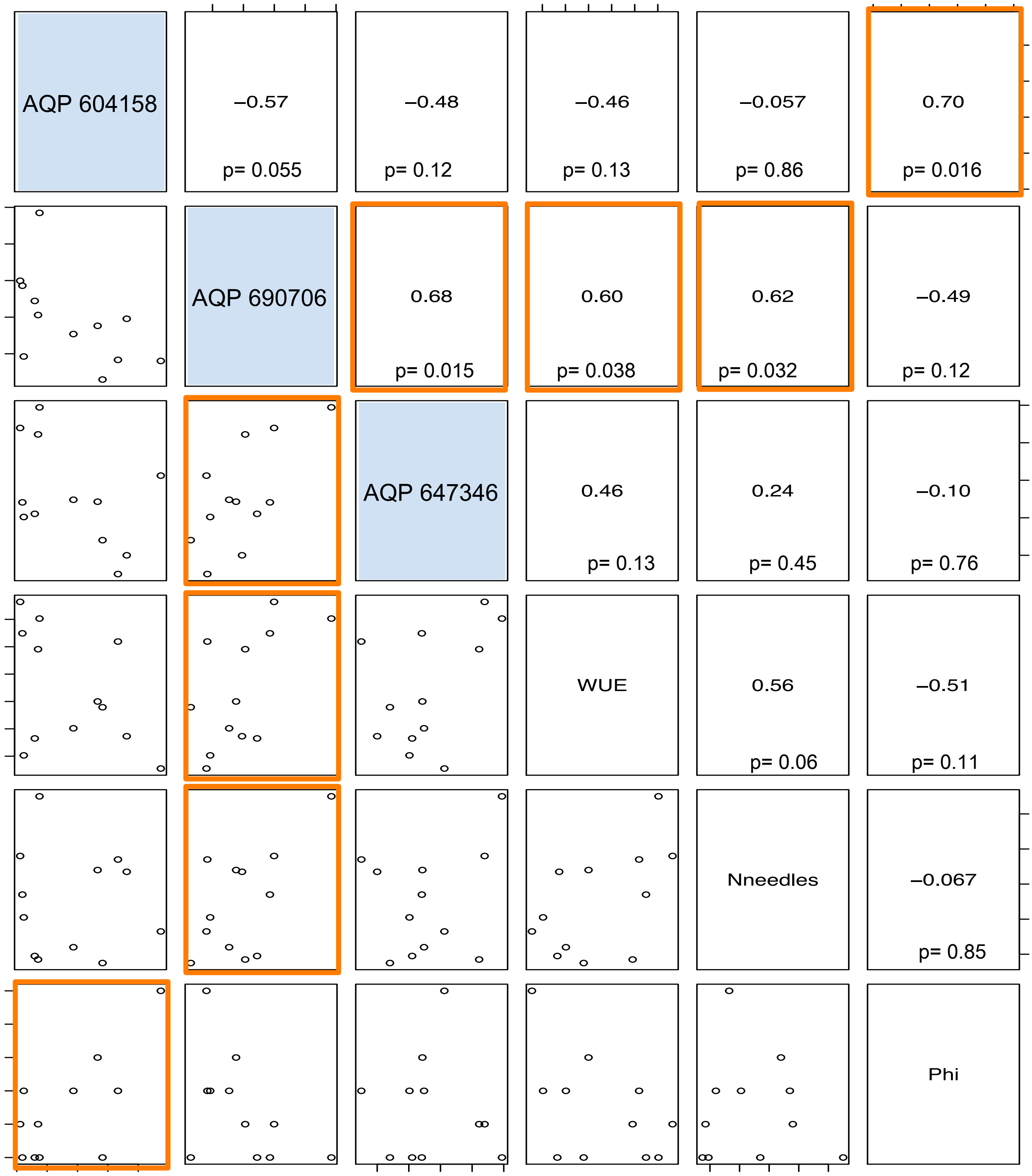

Supplementary Figure 10. Correlation matrix among *C. geophilum* aquaporin gene expression values in mycorrhizas of Scots pine seedlings and parameters of their host plants. The matrix depicts the Pearson correlation coefficient and p-values in the upper triangular and the corresponding scatter plot in the lower triangular. Aquaporin AQP 604158: drought-induced, classical AQP; AQP 690706: symbiosis-induced, classical AQP; AQP 647346: symbiosis-induced aquaglyceroporin. WUE: water use efficiency; Nneedles: nitrogen content in needles; Phi: shoot water potential.

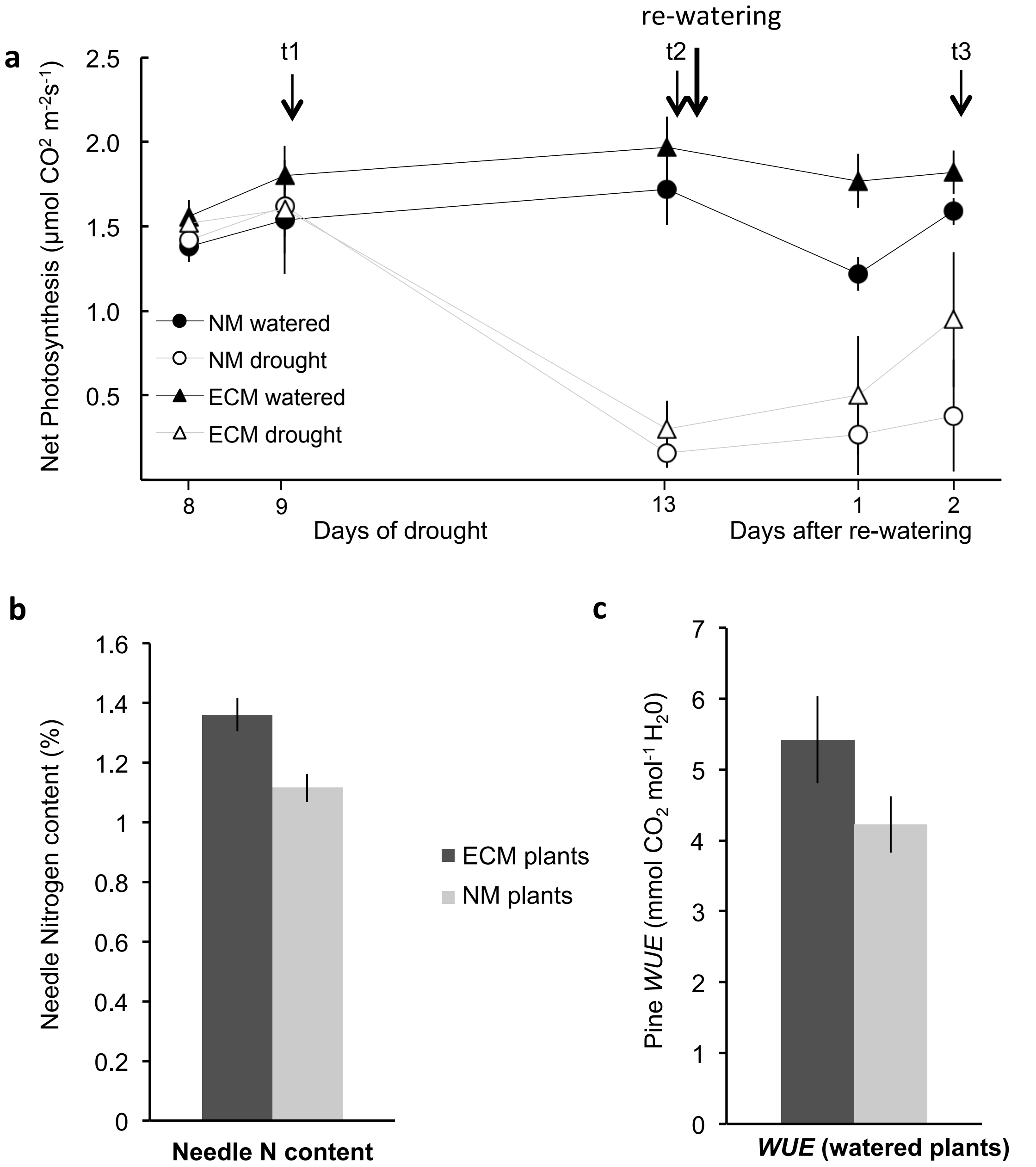

Supplementary Figure 11. Impact of mycorrhization with *Cenococcum geophilum* and of drought and re-watering on *Pinus sylvestris* seedlings. **(a)** Net photosynthesis of mycorrhizal (ECM) and non-mycorrhizal (NM) pine seedlings grown under well-watered conditions or experiencing a drought/re-watering treatment (means of 4 replicates (pots) ±s.e.m.). **(b)** Nitrogen content in needles (means of 24 replicates (pots) ±s.e.m.) and **(c)** Water Use Efficiency (*WUE*) of mycorrhizal (ECM) and non-mycorrhizal (NM) Scots pine seedlings sampled at three timepoints (t1, t2, t3) over the drought/rewatering experiment. For *WUE*, only well-watered (control) plants are considered (means of 12 replicates (pots) ±s.e.m.). Statistics of this data is provided in Supplementary Table 3.

a

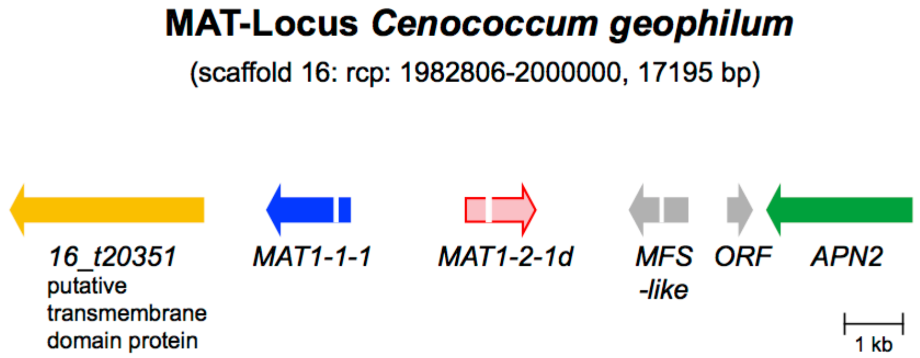

b

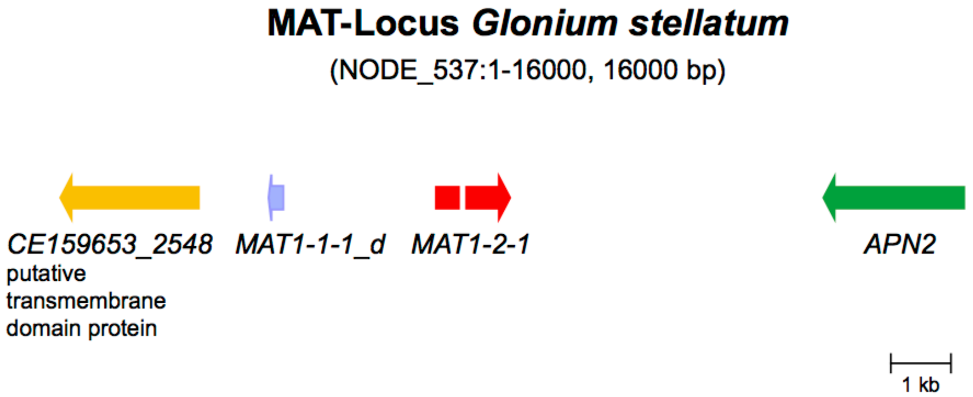

Supplementary Figure 12. Schematic illustration of the mating-type locus *MAT1-1* from *Cenococcum geophilum* and the *MAT1-2* locus from *Glonium stellatum*. (**a**) The positioning and transcriptional direction of the mating-type gene *MAT1-1-1* (blue) of the *C. geophilum MAT1-1* locus and flanking genes are indicated by arrows, *APN2* (green), the putative transmembrane domain gene 16_t20351 (orange), predicted ORFs (grey) and the degenerated *MAT1-2-1* (light red). (**b**) The positioning and transcriptional direction mating-type gene *MAT1-2-1* (red) of the *G. stellatum MAT1-2* locus and flanking genes are indicated by arrows, *APN2* (green), partial putative transmembrane domain gene CE159653_2548 (orange), and the degenerated *MAT1-2-1* sequence (light blue).

Supplementary Table 1: Genome statistics.

Species	Genome assembly size (Mb)	Number of predicted gene models	Sequence coverage depth	Number of scaffolds	Scaffold N50/L50[a] (Mb)	Maximal scaffold size	Nb of gaps	Repeat content (% of genome)
Cenococcum geophilum	177.6	14’748	75	268	26/2.20	6.41	165	81
Glonium stellatum	40.5	14’362	-	2730	395/0.03	0.15	1430	3
Lepidopterella palustris	45.7	13’870	134	3371	242/0.05	0.32	158	15

[a] N50: the smallest number of scaffolds that make up 50% of the genome; L50: the size of the smallest of the L50 scaffolds

Supplementary Table 2. ANOVA tables for data shown in Fig. 5 and Supplementary Fig. 9. a) Impact of drought and rewatering on aquaporin (AQP) expression in *C. geophilum* / *P. sylvestris* ectomycorrhizas (Fig. 5), b) Impact of drought on AQP expression in free-living mycelia (Supplementary Fig. 9a), c) impact of mycorrhization on AQP expression (Supplementary Fig. 9c), as measured by qPCR. Asterisks indicate the significance *p*-value<0.05.

		**AQP Cenge3:647346**	**AQP Cenge3:690706**	**AQP Cenge3:604158**	**AQP Cenge3:689826**	**AQP Cenge3:563409**	**AQP Cenge3:65914[illegible]**
**a)** ANOVA[a]	Drought	$F_{1,18} = 2.65$	**$F_{1,18} = 16.71$***	**$F_{1,18} = 12.38$***	nd	nd	nd
	Timepoint	$F_{2,18} = 1.21$	$F_{2,18} = 0.50$	$F_{2,18} = 2.95$			
	Drought x Timepoint	$F_{2,18} = 1.91$	**$F_{2,18} = 5.97$***	**$F_{2,18} = 8.13$***			
Kruskal Wallis Test for drought effect at single timepoints							
	t 1	H_1 =0.083	H_1 =0.00	H_1 =1.33			
	t 2	**H_1 =5.33***	**H_1 =5.33***	**H_1 =5.33***			
	t 3	H_1 =0.083	**H_1 =5.33***	H_1 =0.00			
**b)** ANOVA	Drought	**$F_{1,10} = 7.63$***	$F_{1,10} = 0.33$	**$F_{1,10} = 13.54$***	$F_{1,10} = 1.27$	$F_{1,10} = 4.60$	$F_{1,10} = 0.79$
	Timepoint	$F_{1,10} = 4.28$	$F_{1,10} = 2.34$	$F_{1,10} = 0.01$	$F_{1,10} = 1.36$	$F_{1,10} = 2.03$	$F_{1,10} = 0.39$
	Drought x Timepoint	$F_{1,10} = 3.16$	$F_{1,10} = 0.21$	$F_{1,10} = 0.01$	$F_{1,10} = 0.77$	$F_{1,10} = 0.48$	$F_{1,10} = 0.59$
**c)** ANOVA	Mycorrhization	**$F_{1,5} = 37.16$***	**$F_{1,5} = 54.14$***	$F_{1,5} = 0.95$	$F_{1,5} = 0.17$	$F_{1,5} = 0.81$	**$F_{1,5} = 11.96$***

[a] Data of Cenge3:604158 were log10 transformed to meet assumption of normality.

Supplementary Table 3. Impact of drought and re-watering on one-year-old *Pinus sylvestris* seedlings mycorrhizal or not with *C. geophilum*. Net photosynthesis (*A*; µmol CO_2 m^{-2} s^{-1}), stomatal conductance (*gs*; mol H_2O m^{-2} s^{-1}), water use efficiency (*WUE*; A/transpiration; mmol CO_2 mol^{-1} H_20), pre-dawn water potential of shoots (Ψ; MPa), needle N concentration (%) dry weight (DW; g) of whole shoots and roots of mycorrhizal (ECM) and nonmycorrhizal (NM) *Pinus sylvestris* seedlings under drought-stressed (drought) or well-watered (watered) conditions. Destructive measurements (Ψ, N needles, DW) were performed at 10 and 14 days of drought and 3 days after rewatering. Non-destructive plant gas exchange measurements were performed the day before harvesting and additionally at 8 days of drought and 1 day after re-watering. Numbers are means of 4 replicates ±s.e.m.

			A (µmol m^{-2} s^{-1})	***gs*** (mol H_2O m^{-2} s^{-1})	***WUE*** (mmol CO_2 mol^{-1} H_2O)	**Ψ shoot** (MPa)	**N needles** (%)	**DW shoot** (g)	**DW roots** (g)
drought	day 8	NM watered	1.38 ±0.09	0.021 ±0.002	2.7 ±0.1	nd[d]	nd	nd	nd
		NM drought	1.42 ±0.08	0.017 ±0.002	3.4 ±0.2				
		ECM watered	1.56 ±0.03	0.019 ±0.001	3.5 ±0.3				
		ECM drought	1.52 ±0.14	0.020 ±0.001	3.6 ±0.1				
	day 9/10	NM watered	1.54 ±0.20	0.018 ±0.002	3.3 ±0.3	-0.8 ±0.1	1.27 ±0.19	0.53 ±0.06	0.73 ±0.06
		NM drought	1.62 ±0.18	0.013 ±0.002	4.7 ±0.3	-0.9 ±0.1	1.01 ±0.03	0.51 ±0.09	0.80 ±0.06
		ECM watered	1.80 ±0.09	0.017 ±0.002	4.5 ±0.8	-0.8 ±0.3	1.32 ±0.12	0.57 ±0.03	0.69 ±0.06
		ECM drought	1.60 ±0.38	0.016 ±0.004	4.2 ±0.5	-1.0 ±0.3	1.43 ±0.05	0.55 ±0.06	0.78 ±0.05
	day 13/14	NM watered	1.72 ±0.21	0.018 ±0.001	3.6 ±0.5	-0.7 ±0.1	0.91 ±0.05	0.49 ±0.05	0.82 ±0.06
		NM drought	0.16 ±0.09	0.000 ±0.000	nc[b]	-3.6 ±0.4	1.14 ±0.08	0.54 ±0.09	0.72 ±0.04
		ECM watered	1.97 ±0.18	0.018 ±0.001	4.4 ±0.8	-0.5 ±0.1	1.25 ±0.12	0.49 ±0.05	0.65 ±0.06
		ECM drought	0.30 ±0.17	0.001 ±0.001	nc	-3.6 ±0.2	1.35 ±0.11	0.67 ±0.03	0.96 ±0.10
after re-watering	day 1	NM watered	1.22 ±0.10	0.016 ±0.001	2.9 ±0.1	nd	nd	nd	nd
		NM drought	0.27 ±0.24	0.001 ±0.001	nc				
		ECM watered	1.77 ±0.16	0.015 ±0.002	5.5 ±1.6				
		ECM drought	0.50 ±0.35	0.004 ±0.003	nc				
	day 2/3	NM watered	1.59 ±0.08	0.013 ±0.000	5.1 ±0.2	-0.4 ±0.0	1.10 ±0.11	0.53 ±0.01	0.83 ±0.03
		NM drought	0.38 ±0.33	0.001 ±0.001	nc	-0.2 ±0.1	1.26 ±0.11	0.51 ±0.03	0.64 ±0.07
		ECM watered	1.82 ±0.13	0.013 ±0.001	6.2 ±0.7	-0.5 ±0.0	1.33 ±0.23	0.61 ±0.11	0.83 ±0.10
		ECM drought	0.95 ±0.40	0.005 ±0.003	nc	-0.3 ±0.1	1.53 ±0.18	0.63 ±0.07	0.79 ±0.10

continued

Supplementary Table 3 continued

		A ($\mu mol\ m^{-2}\ s^{-1}$)	***gs*** ($mol\ H_2O\ m^{-2}\ s^{-1}$)	***WUE*** ($mmol\ CO_2\ mol^{-1}\ H_2O$)	**Ψ shoot** (MPa)	**N needles** (%)	**DW shoot** (g)	**DW roots** (g)
**Statistics**	Effect	REML [a]	REML	REML[c]	3-way ANOVA	2-way ANOVA	2-way ANOVA	2-way ANOVA
	Mycorrhization	**$F_{1,48}$ = 5.40***	$F_{1,47}$ = 0.21	**$F_{1,26}$ = 8.11***	$F_{1,34}$ = 0.41	**$F_{1,44}$ = 11.48***	$F_{1,44}$ = 3.87	$F_{1,44}$ = 0.40
	Drought	**$F_{1,48}$ = 50.88***	**$F_{1,47}$ = 82.69***		**$F_{1,34}$ = 85.33***	$F_{1,44}$ = 1.42	$F_{1,44}$ = 0.77	$F_{1,44}$ = 0.27
	Day	**$F_{4,26}$ = 8.00***	**$F_{4,28}$ = 14.34***	**$F_{4,13}$ = 8.71***	**$F_{2,34}$ = 110.00***			
	Mycorrhization x Drought	$F_{1,47}$ = 0.19	$F_{1,47}$ = 1.29		$F_{1,34}$ = 0.19	$F_{1,44}$ = 0.35	$F_{1,44}$ = 0.55	**$F_{1,44}$ = 5.24***
	Drought x Day	**$F_{4,26}$ = 18.03***	**$F_{4,28}$ = 11.34***		**$F_{2,34}$ = 102.81***			

[a] REML: Linear mixed effects model using the restricted maximum likelihood (REML) estimation method in which repeated measurements at some days are taken into account as random factor.
[b] WUE: nc = not calculable because transpiration was not measurable (values at 0).
[c] In this analysis, only watered (control) samples were taken into account.
[d] na = not assessed
* p<0.05

Supplementary References

1. Floudas, D. *et al.* The Paleozoic Origin of Enzymatic Lignin Decomposition Reconstructed from 31 Fungal Genomes. *Science* **336**, 1715-1719 (2012).

2. Kohler, A. *et al.* Convergent losses of decay mechanisms and rapid turnover of symbiosis genes in mycorrhizal mutualists. *Nat. Genet.* **47**, 410-415 (2015).
